# Clinical Outcomes and Donor-specific Antibody Rebound 5 y After Kidney Transplant Enabled by Imlifidase Desensitization

**DOI:** 10.1097/TXD.0000000000001752

**Published:** 2025-01-09

**Authors:** Ian S. Jaffe, Anna Runström, Vasishta S. Tatapudi, Elaina P. Weldon, Cecilia L. Deterville, Rebecca A. Dieter, Robert A. Montgomery, Bonnie E. Lonze, Massimo Mangiola

**Affiliations:** 1 Department of Surgery, New York University Grossman School of Medicine, New York, NY.; 2 New York University Langone Transplant Institute, New York, NY.; 3 Hansa Biopharma, Lund, Sweden.; 4 Department of Medicine, New York University Grossman School of Medicine, New York, NY.; 5 Department of Pathology, New York University Grossman School of Medicine, New York, NY.

## Abstract

**Background.:**

Imlifidase is an IgG-cleaving endopeptidase conditionally approved in Europe for desensitization of highly sensitized patients before kidney transplantation. We present 5-y outcomes and donor-specific antibody (DSA) levels for clinical trial participants from a single site who received imlifidase for desensitization before incompatible transplantation (NCT02790437).

**Methods.:**

Imlifidase was administered up to 24 h before living or deceased donor kidney transplantation. DSAs were monitored before transplantation, at days 7 and 28, and at 5 y posttransplant.

**Results.:**

At 5 y, 7 of 8 participants were alive. One of these 7 had suboptimal graft function secondary to donor-derived disease but remained dialysis independent. Three participants had antibody-mediated rejection (AMR), which occurred in the first 30 d in all cases and was successfully treated. No new episodes of suspected or biopsy-proven AMR occurred after 30 d posttransplant. Seven participants had DSA rebound. DSAs commonly persisted 5 y posttransplant, although they were generally lower strength compared with pre-imlifidase. Dilution studies of sensitized serum enabled the identification of lower AMR risk phenotypes for persisting DSAs. Severe and/or opportunistic infections were not observed at greater than expected frequency.

**Conclusions.:**

Five-year outcomes of imlifidase-enabled incompatible transplants are overall favorable. DSA rebound is common, but antibody strength lessens in the long term, and longitudinally persisting DSAs did not lead to premature graft failure.

## Graphical abstract

**Figure:**
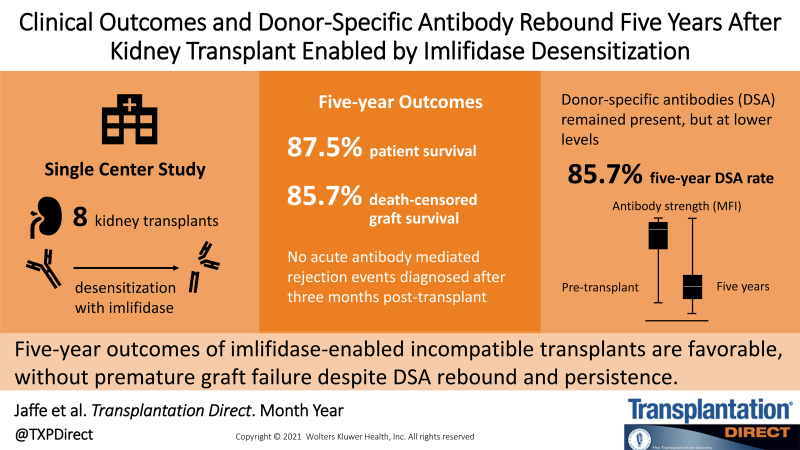

Kidney transplant candidates previously exposed to alloantigens often develop anti-HLA antibodies. When specific to a potential kidney donor’s HLA epitopes, these donor-specific antibodies (DSAs) can cause hyperacute, acute, and/or chronic antibody-mediated allograft rejection (AMR).^1-3^ Safe transplantation in the setting of preexisting high-strength DSA requires desensitization, whereby antibodies are removed from the recipient’s circulation before transplantation. Plasma exchange is the most common desensitization modality in the United States,^4^ but is inefficient and requires multiple treatments over days to weeks to reduce high-strength DSA. Thus, desensitization is practically only feasible for patients with living donors. Despite allocation system priority given to the most highly sensitized patients, designed to increase chances of receiving a compatible deceased donor offer, patients with calculated panel-reactive antibody (cPRA) >99.9% have rates of transplantation that approach zero.^5-8^ For these patients, desensitization and incompatible transplantation afford a clear survival benefit over waiting for a compatible offer that may never materialize.^9^

Imlifidase is a *Streptococcus pyogenese*–derived cysteine protease that cleaves human IgG at the hinge region, effectively eliminating all complement-fixing functions and preventing hyperacute rejection.^10,11^ Imlifidase also cleaves the B-cell receptor, which may temporarily prevent memory B-cell activation.^12^ This novel drug rapidly eliminates all circulating IgG in approximately 4–6 h,^10^ and therefore, it offers a means of desensitization that can be used for recipients of both living and deceased donor incompatible kidneys. Phase II clinical trials in which patients received kidney transplants after imlifidase desensitization have demonstrated both safety and efficacy.^13-15^ Early acute AMR was observed in 20% of participants, but in a cohort of patients followed out to 3 y, no occurrences of AMR were observed after 6 mo.^16^ Patient survival and death-censored allograft survival at 3 y were 90% and 84%, respectively.^16^ Importantly, 84% of the patients enrolled in these studies received kidneys from deceased donors, transplants not likely to have been possible with traditional desensitization methods.^13,14^

Imlifidase has been conditionally approved by the European Medicines Agency^17^; however, the drug remains investigational in the United States. A recently reported randomized trial comparing imlifidase to plasma exchange in the treatment of AMR demonstrated that despite significantly greater efficacy in reducing DSA, imlifidase-treated patients did not have improved graft function.^18^ In desensitization, a multicenter phase III randomized controlled trial currently underway (NCT04935177) is designed to compare imlifidase to the currently available standard care treatment options. Alongside this trial—which will provide currently lacking head-to-head efficacy data in desensitization—it is imperative to understand the long-term outcomes of patients treated with this novel drug. Specifically, examining whether pretransplant DSA strength and/or specificity can predict their longitudinal behavior could help inform organ acceptance decisions for future patients. In this study, we report clinical outcomes and DSA levels 5 y after imlifidase-enabled incompatible kidney transplantation for 8 participants who were previously treated at our site.

## PATIENTS AND METHODS

### Patient Population and Regulatory Oversight

The participants reported here were transplanted as part of an open-label single-arm phase II study of imlifidase (NCT02790437) under an investigational new drug application held by Hansa Biopharma (Lund, Sweden). The initial clinical trial, as well as a separate observational follow-up study, was approved by the New York University Grossman School of Medicine Institutional Review Board. The study design as well as early outcomes for 7 of 8 participants has been previously reported.^15^ In brief, participants were desensitized with imlifidase before receiving an incompatible transplant from either a living or deceased donor. A pre-imlifidase-positive crossmatch by flow cytometric and/or complement-dependent cytotoxicity assay was required for inclusion in the original clinical trial. Participant 8 was desensitized under the same protocol.

### Imlifidase Desensitization and Immunosuppression Management

Imlifidase (0.25 mg/kg intravenously) was administered upon availability of an incompatible donor kidney as previously described.^15^ T cell–depleting antibody induction therapy (alemtuzumab) was administered in a delayed manner (postoperative day [POD] 4) due to the susceptibility of this humanized antibody to cleavage by imlifidase. Corticosteroid pulse/taper and mycophenolate (2 g total daily dose) were initiated at the time of transplantation. Maintenance immunosuppression consisted of standard triple therapy with tacrolimus (trough goal 8–10 ng/mL), mycophenolate (2 g total daily dose), and prednisone (5 mg). For one participant (participant 4), tacrolimus was converted to belatacept at 3.7 mo posttransplant.

### Clinical Outcome Measures

Clinical outcomes assessed through 5 y posttransplantation included patient survival, graft survival, graft function, occurrence of AMR, occurrence of major infections, and cytomegalovirus (CMV) or BK virus viremia. Graft loss was defined as either requiring maintenance dialysis or retransplantation. Graft function was evaluated using serum creatinine and estimated glomerular filtration rate (eGFR) using the Chronic Kidney Disease Epidemiology Collaboration 2021 equation.^19^ AMR was diagnosed using the most recent Banff criteria at the time of biopsy.^20^ Major infections were defined as either a hospitalization where the primary cause for admission was a bacterial, viral, fungal, or parasitic illness or a hospitalization that was prolonged because of an infectious process. Clinically significant CMV and BK infection was defined as any occurrence of quantifiable viremia, regardless of treatment. Total IgG levels measured for routine clinical care were also collected.

### Assessment of HLA Antibodies

Low- or intermediate-resolution HLA typing was available for all donor-recipient pairs as per standard United Network for Organ Sharing requirements. For patients with previous transplants, previous donor HLA typing was acquired when available to identify repeated HLA mismatches. DSAs were defined at the antigen/split-level resolution.

HLA antibody screening was performed with single-antigen beads on the Luminex platform (One Lambda, Thermo Fisher West Hills, CA). Sera were pretreated with EDTA to prevent signal inhibition. Mean fluorescence intensity (MFI) >2000 was used as the threshold of positivity.^21^ Whenever sufficient serum was available, serum dilutions of 1:16, 1:32, 1:64, and 1:128 were tested by Luminex to predict the likelihood of complement fixation. An antibody was considered capable of activating complement if serum diluted 1:16 yielded an MFI of ≥8000. Antibody titer was defined as the highest dilution at which MFI was ≥8000. Dilution testing was performed on serum samples collected pretransplant, 7 d, 28 d, and 5 y posttransplant provided adequate serum was available. Samples collected at other time points for clinical indication were also evaluated. For HLA antibodies represented by multiple beads, the median MFI and SD were reported. Otherwise, the MFI of the highest allele-specific DSA bead was reported. A DSA was considered to be predictive of a positive flow cytometric crossmatch (FCXM) if individual or cumulative HLA-A/B/DR/DQ was >5000 MFI and/or >10 000 MFI for individual or cumulative HLA-C/DP.^22^ Based on prior published work, a DSA was considered to be high-risk for AMR if the MFI value was >8000 in both neat serum and 1:16 dilution or <4000 in neat serum and >8000 in 1:16 dilution.^23^

### Analytical Methods

Descriptive analyses are reported for clinical outcomes and antibody data. DSAs, mean third-party HLA antibody levels, and total IgG levels are reported individually and in aggregate. In aggregate measures, where specified, DSAs were restricted to only the immunodominant DSAs to minimize the influence of participants with multiple DSAs. Pretransplant and year 5 levels were compared using paired *t* tests. All analyses were performed using GraphPad Prism 10.0.2 (Boston, MA).

## RESULTS

### Patient Demographics

Clinical and HLA antibody data were collected through 5 y posttransplant for 7 participants and until the time of death for 1 participant. The complete demographics of 7 of the 8 recipients discussed herein have been previously reported.^15^ Demographics and clinical data for the eighth participant (as well as all recipients discussed in this report) are detailed in **Supplemental Materials** (**SDC,** http://links.lww.com/TXD/A733; clinical summary of participants). Briefly, the average age at transplant was 41 y (SD = 11), 63% were men, and the median time on dialysis was 7.0 y (interquartile range, 1.7–8.7; Table 1). All had a cPRA ≥96%, with a median cPRA of 99%. Seven of 8 participants had received prior kidney transplant(s). Five participants received deceased donor kidneys (all from brain-dead donors) and 3 received living donor kidneys. Detailed participant-level descriptions and results are available in **Supplemental Materials** (**SDC,** http://links.lww.com/TXD/A733; clinical summary of participants) and **Figures S1–S16** (**SDC,** http://links.lww.com/TXD/A733).

**TABLE 1.** Clinical outcomes 5 y after imlifidase-enabled kidney transplant

Participant	Age/sex	Total dialysis, y	Cause of ESKD	Prior kidney transplant	cPRA	Repeat HLA mismatch	5-y Status	5-y eGFR (mL/min/1.73 m^2^)	Donor	Early AMR^*a*^	Late AMR^*b*^	Major infection^*c*^
1	64/male	9.1	Unknown	1	100	Unknown	Deceased	–	DBD	No	No	Yes
2	45/female	23.3	PKD	0	100	–	Alive with a functioning graft	34	DBD	Yes	No	Never
3	31/male	7.3	Unknown	1	99	A24	Alive with poor graft function	21	DBD	Yes	No	Yes
4	40/male	4.5	DM	1	100	Unknown	Alive with a functioning graft	23	DBD	Yes	No	Yes
5	31/female	7.4	SLE	2	98	None	Alive with a functioning graft	85	DBD	No	No	Yes
6	33/male	6.6	CRN	3	99	DR12	Alive with a functioning graft	54	Living	No	No	Yes
7	42/female	0.8	HTN	1	100	DQ5	Alive with a functioning graft	51	Living	No	No	Never
8	39/male	0.7	Unknown	1	96	None	Alive with a functioning graft	42	Living	No	No	Never

^*a*^AMR before 3 mo.

^*b*^AMR after 3 mo.

^*c*^Major infection was defined as an infection requiring hospitalization.

AMR, antibody-mediated rejection; cPRA, calculated panel-reactive antigen; CRN, congenital reflux nephropathy; DBD, donation after brain death; DM, diabetes mellitus; eGFR, estimated glomerular filtration rate; ESKD, end-stage kidney disease; HTN, hypertension; PKD, polycystic kidney disease; SLE, systemic lupus erythematosus.

### Clinical Outcomes

Seven of 8 participants were alive 5 y posttransplant. One participant died from influenza infection complicated by sepsis 12 mo posttransplant. Of 7 participants alive at 5 y posttransplant, 6 had well-functioning grafts. One participant (participant 3) was relisted for transplant 36 mo posttransplant due to graft dysfunction from donor-derived vascular disease and fibrosis (evident on a biopsy 2 wk posttransplant but not appreciated pretransplant) but remained off dialysis through 5-y follow-up (see **Supplemental Materials, SDC,** http://links.lww.com/TXD/A733). As previously reported, 3 participants had acute AMR, in all cases occurring within the first month posttransplant and rescued with standard treatments.^15^ No participants had any episodes of AMR beyond the first 30 days. Five participants had major infections over the 5 y of follow-up. Four participants developed CMV viremia, which in all cases resolved with valganciclovir; 1 participant developed brief BK viremia. The mean eGFR at 5 y among alive patients was 42.3 mL/min/1.73 m^2^ (SD = ±22; subject-level eGFR over time is shown in **Figure S17** (**SDC,** http://links.lww.com/TXD/A733). Additional details on the timing and treatment of significant infections are provided in **Supplemental Materials** (**SDC,** http://links.lww.com/TXD/A733).

### Imlifidase Effect and Early DSA Rebound

Following imlifidase administration, all participants had near elimination of all HLA antibodies. Predose, median composite MFI among all DSAs of all patients was 20 520 (SD = 7474) in neat samples. At 24 h postdose, this was decreased to 1261 (SD = 1196). Three of the 8 participants had early DSA rebound and acute AMR, which in all cases onset within the first 30 d posttransplant and have been previously reported.^15^ These AMR cases corresponded with rapidly rebounding DSAs, which were observed as part of the original phase II study.

In this series, AMR was caused by the rebound of different DSAs to varying strengths. For example, participant 2 (who was broadly sensitized because of repeated blood transfusions and expressed 5 class I DSAs and 1 class II DSA) had an early (POD9) AMR associated with rebound of multiple DSAs (anti-A1 to peak neat MFI = 24 738 on POD11, anti-B8 to MFI = 5125 on POD9, anti-cw9 to MFI = 11 560 on POD11, anti-cw12 to MFI = 9071 on POD9, and anti-DQ6 to MFI = 8203 on POD9). Participant 3, meanwhile, experienced acute AMR associated with a single class II DSA rebound (DQ4, which reached a peak neat MFI of 18 043 on POD24). Similarly, participant 4 also had acute AMR associated with early class II DSA rebound (DP1 to peak neat MFI = 17 922 on POD9 and DP4 to MFI = 23 175 on POD10).

### Long-term Class I DSAs

**FIGURE 1.**
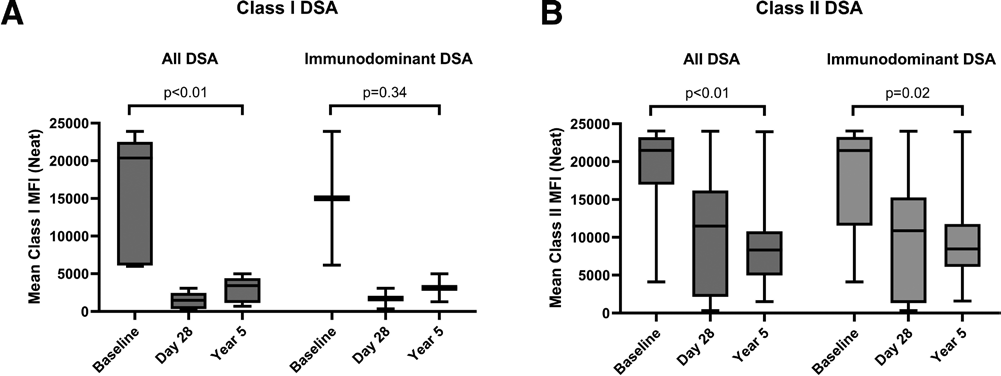
Aggregate DSA levels stratified by HLA class. A, The distribution of all class I DSA MFIs over time (left) and the distribution of only the immunodominant class I DSAs (right) over time. Box upper and lower bounds are at the third and first quartiles, with the median value annotated and the line bounding the range of values. Baseline and year 5 were compared using a paired *t* test. B, The distribution of all class II DSA MFIs over time (left) and the distribution of only the immunodominant class II DSAs (right) over time, with the same schema. DSA, donor-specific antibody; MFI, mean fluorescence intensity.

**FIGURE 2.**
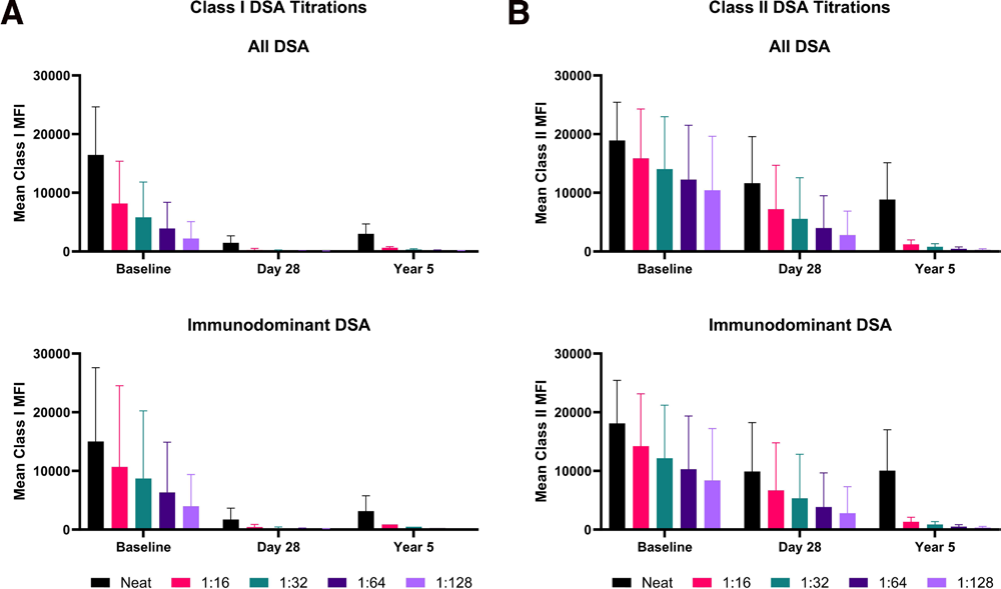
Aggregates of dilutional titrations for DSA strength. Sera were serially diluted up to 1:128 concentration and separately run on Luminex single-antigen beads. DSAs were identified on the basis of patient and donor HLA typing. A, HLA class I DSA titrations. B, HLA class II DSA titrations. In both cases, the upper panel shows the average MFI when all DSAs were included, whereas the lower panel shows the average MFI in each serial dilution restricted only to the immunodominant DSA to mitigate the disproportionate impact of participants with multiple DSAs. DSA, donor-specific antibody; MFI, mean fluorescence intensity.

Two of the 8 participants expressed at least 1 HLA class I DSA (Figure 1A). Compared with baseline (mean MFI = 16 465), class I DSAs were weaker at day 28 (mean MFI = 1483) and remained significantly below baseline 5 y posttransplant (mean MFI = 3009; *P* < 0.01). A similar pattern was recapitulated when analysis was restricted to the immunodominant DSAs for each participant: baseline mean MFI of 15 023 versus 1723 at day 28 and 3136 at 5 y (*P* = 0.36 for baseline versus year 5). This durable weakening of class I DSA strength was also qualitatively confirmed in dilutional titrations, where class I DSAs were all at an MFI <1000 in 1:16 dilution at day 28 and year 5 (Figure 2A).

### Long-term Class II DSA

Unlike class I DSAs, all participants exhibited at least 1 class II DSA. From a baseline mean MFI of 18 914 pretransplant, class II DSAs were weaker at day 28 (mean MFI = 11 640) and year 5 (mean MFI = 8078; *P* < 0.01; Figure 1B). This pattern remained true when restricted to immunodominant DSAs only: baseline mean MFI of 18 084 versus 9908 at day 28 and 8870 at 5 y (*P* = 0.02 for baseline versus year 5). For 7 of the 8 participants, DSA levels were predictive of a positive FCXM at some point after transplant (Table 2). At 5 y, 6 of the 7 living participants had DSA levels predictive of a positive FCXM. Although DSAs appeared to be approximately equal in strength at day 28 and year 5, DSA titrations revealed that year 5 DSAs had notably reduced strength compared with day 28 (and pretransplant baseline) DSAs. Although 8 of 11 DSAs (73%; and 5/8 immunodominant DSAs) had an MFI >2000 in 1:16 dilution at day 28, none met this threshold at year 5, which is consistent with significantly weaker DSAs. Correspondingly, none of these DSAs met the criteria for a high AMR risk DSA (high titer, likely complement-activating) in the 4 cases where DSA titration could be performed.

**TABLE 2.** Longitudinal interpretation of DSA levels and association with acute AMR

				Predicted flow cytometry crossmatch^*a*^					
Participant	No. of DSAs	Class of DSAs	Repeat HLA mismatch	Baseline	Day 7	Day 28	5 y	DSA high risk titer^*b*^ at 5 y	Beginning of AMR treatment
1	1	II	Unknown	**+**	**+**	**+**	N/A	NA	No AMR
2	6	I/II	None	**+**	**–**	**–** ^*c*^	+^*d*^	**–**	Day 9
3	2	I/II	A24	**+**	**+**	+^*c*^	**+**	NA	Day 26
4	2	II	Unknown	**+**	+^*c*^	**+**	**+**	NA	Day 6
5	1	II	None	**+**	**–**	**–**	**+**	**–**	No AMR
6	1	II	DR12	**+**	**–**	**–**	**–**	NA	No AMR
7	2	II	DQ5	**+**	**+**	**+**	**+**	**–**	No AMR
8	2	II	None	**+**	**+**	**+**	**+**	**–**	No AMR

^*a*^Flow crossmatch predicted as positive if cumulative HLA-A/B/DR/DQ >5000 MFI and/or cumulative HLA-C/DP >10 000 MFI.

^*b*^High-risk titer based on MFI >8000 for any DSA at 1:16 dilution, adapted from Zeevi and Lunz.^23^

^*c*^Drawn within 48 h after plasmapheresis.

^*d*^Postfunctional splenectomy.

AMR, antibody-mediated rejection; DSA, donor-specific antibody; MFI, mean fluorescence intensity.

### Effect of Repeated HLA Mismatches

**FIGURE 3.**
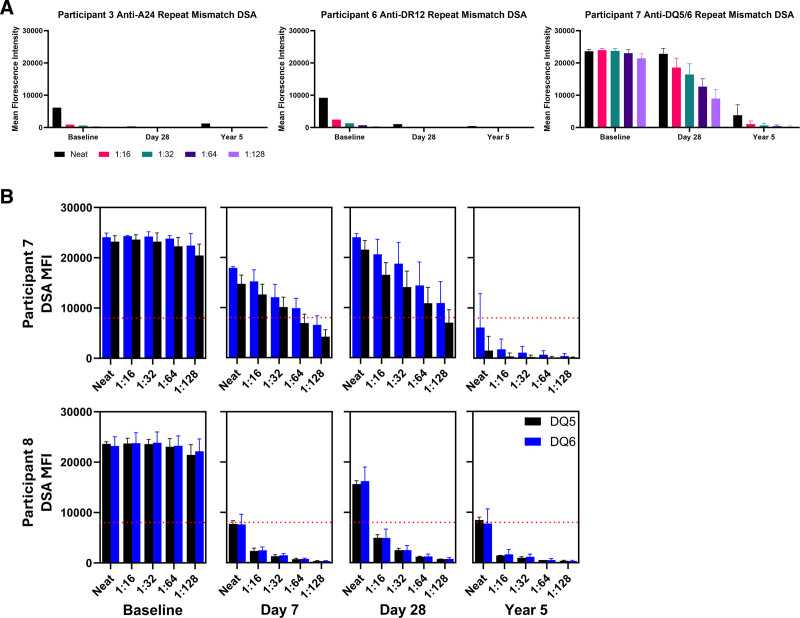
Donor-specific antibody titrations for repeat HLA mismatches. A, DSA titrations for the 3 cases of confirmed repeat HLA mismatches. B, A comparison between participants 7 and 8, who produced the same DSAs against a common epitope on DQ5 and DQ6 with comparable baseline strength. DSAs of participant 7 was a case of repeat HLA mismatch, whereas DSAs of participant 8 was not. DSA, donor-specific antibody; MFI, mean fluorescence intensity.

HLA typing for a prior kidney transplant was available for 6 of 8 participants. Three participants had confirmed repeat HLA mismatches with a prior kidney donor based on these data. These included a repeat class I DSA (anti-A24 for participant 3) and 2 class II DSAs (anti-DR12 for participant 6 and a DSA against a common epitope on DQ5 and DQ6 for participant 7). In the cases of the anti-A24 and anti-DR12 repeat mismatch DSAs, both had relatively weak baseline strengths with a pretransplant MFI <10 000 and never rebounded (Figure 3A). However, in the case of participant 7 DSAs against DQ5/6, a strong pretransplant level (mean MFI = 23 638) subsequently corresponded to a strong rebound at day 7 (mean MFI = 16 329) and day 28 (mean MFI = 22 801), although there was a weakening in the strength of this DSA by 5 y posttransplant (mean MFI = 3808).

Interestingly, participant 8 and participant 7 both produced a DSA against a common epitope on DQ5 and DQ6 at comparable baseline strengths (fully saturating to >20 000 MFI even at 1:128 dilution at baseline; Figure 3B). However, while DSAs of participant 7 were a repeat HLA mismatch, DSAs of participant 8 were not. Although DSAs of participant 7 had a much stronger initial rebound in the first month posttransplant than participant 8 (>8000 MFI even to 1:128 dilution on day 28 versus dropping below an MFI of 800 at 1:16 dilution at the same time point), the opposite was true for long-term DSA strength (although both DSAs were qualitatively weak, with 1:16 titrations <2000 MFI at year 5).

### Total IgG and Third-party Antibodies

**FIGURE 4.**
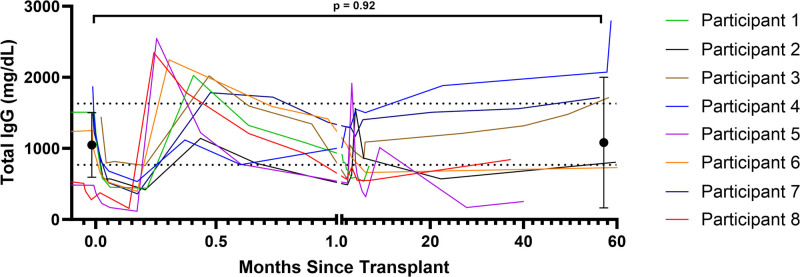
Total IgG levels pre- and posttransplant. Total IgG levels were monitored as part of the original study protocol and then as part of routine clinical care. Pretransplant and the latest total IgG level through the 5 y of follow-up were compared using a paired *t* test.

**FIGURE 5.**
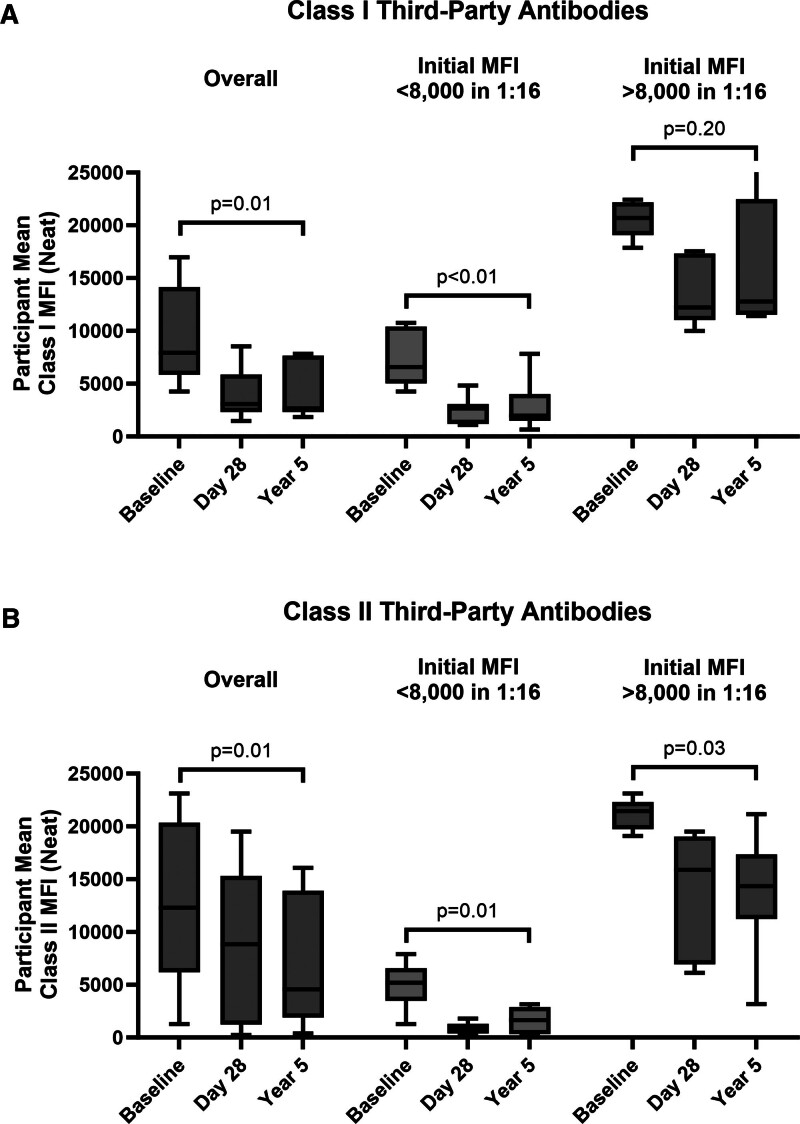
Aggregate third-party antibody levels stratified by HLA class. A, The distribution of all class I third-party MFIs over time (left) and stratified by baseline weak third-party antibodies (middle; pretransplant MFI <8000 in 1:16 dilution) or baseline strong third-party antibodies (right; pretransplant MFI >8000 in 1:16 dilution). Box upper and lower bounds are at the third and first quartiles, with the median value annotated and the line bounding the range of values. Baseline and year 5 were compared using a paired *t* test. B, The distribution of all class II third-party MFIs over time (left) and stratified by baseline weak third-party antibodies (middle; pretransplant MFI <8000 in 1:16 dilution) or baseline strong third-party antibodies (right; pretransplant MFI >8000 in 1:16 dilution), with the same schema. DSA, donor-specific antibody; MFI, mean fluorescence intensity.

IgG levels were generally reconstituted within 2 wk and did not significantly differ from pretransplant (mean = 1188 mg/dL) to several years posttransplant (mean = 1217 mg/dL; *P* = 0.92; Figure 4). However, HLA class I third-party antibodies remained significantly lower than baseline (mean MFI = 9353) at 5 y posttransplant (mean MFI = 4181, *P* < 0.01; Figure 5). Both weak (baseline MFI <8000 at 1:16 dilution) and strong class I (baseline MFI >8000 at 1:16 dilution) third-party antibodies remained below their initial titer (*P* < 0.01 and *P* = 0.20, respectively), and with weaker titrations (**Figure S18A, SDC,** http://links.lww.com/TXD/A733). Similarly, HLA class II third-party antibodies remained significantly lower than baseline (mean MFI = 12 849) at 5 y posttransplant (mean MFI = 6540, *P* = 0.01) and demonstrated the same pattern of remaining lower regardless of baseline titer (*P* < 0.01 and *P* = 0.03, respectively) and with weaker titrations (**Figure S18B, SDC,** http://links.lww.com/TXD/A733).

## DISCUSSION

We report here a single-center cohort of imlifidase-desensitized patients who have reached 5 y posttransplant, demonstrating an overall 5-y survival rate of 87.5% and a death-censored 5-y graft survival rate of 85.7%. We also extend prior findings^16^ that while some patients desensitized with imlifidase experienced treatable AMR in the first few months posttransplant, AMR beyond the first few months appears to be rare (0% beyond 30 d through 5 y posttransplant in our cohort), despite the persistence of some DSA. Participants with functioning grafts showed very stable eGFR through 5 y, with no drops in eGFR concerning impending late allograft failure.^24^

The long-term impact of DSA persistence is an important concern for patients who undergo desensitization, given the higher AMR risk in this population.^25^ It has been previously established that early acute AMR in imlifidase-desensitized patients is associated with higher pretransplant and rebound MFIs, but the starting antibody strength thresholds above which AMR is most often observed remain undefined.^16^ Similarly, there is concern from non-imlifidase cohorts that persistent DSAs in the setting of a well-functioning graft pose a risk for subclinical or chronic AMR that portends a worse long-term graft prognosis.^26^

Both class I and class II DSA rebound contributed to acute AMR in this cohort. Notably, only 2 participants in this study had HLA class I DSAs, and in neither case was there any significant long-term persistence of the DSAs. Class II DSAs behaved more heterogeneously long-term, although the DSAs that persisted were near-universally weakened. Some participants had minimal rebound and persistence of class II DSAs. It is possible that the concomitant treatment with IVIG and rituximab is responsible for this minimal rebound, although prior studies of desensitization with combined IVIG and rituximab alone have demonstrated a much more minimal DSA rebound than seen in our cohort.^27,28^ This may be because desensitization with IVIG and rituximab alone is more likely to be successful in individuals with lower-strength DSAs, presumably at lower risk for significant rebound. At the same time, some participants had a long-term decrease in DSA levels after an initial rebound, which was also the overall pattern observed in the cohort.

In our cohort, all but 1 patient was previously transplanted and up to 5 participants (for 2 participants, the HLA type of the immunizer was unknown) underwent transplant against a repeat HLA mismatch (1 class I, 2 class II, and 2 unknowns with possible class II). The DSAs corresponding to these repeat HLA mismatches took disparate courses: some were completely quiescent and never rebounded (anti-DR12 for participant 6 and anti-A24 for participant 3), whereas 1 participant (participant 7) had one of the strongest DSA rebounds observed in the cohort (repeat against DQ5/DQ6). This difference qualitatively correlated with pretransplant baseline DSA strength, which was <10 000 MFI and low titer (<1:16) for participants 6 and 3, but >20 000 MFI and high titer (>1:128) for participant 7. However, it is also possible that DSAs linked to repeat HLA mismatch in our analysis may only represent coincidental antigen-level repeat mismatches. It is possible that some of the DSAs identified in our analysis as repeats may represent allele and antigen-level specificity against the current donor but only coincidental antigen-level but not allelic-level specificity against the prior immunizer, representing a spurious repeat mismatch. Unfortunately, we were unable to analyze these data in detail given the inconsistent availability of intermediate- or high-resolution HLA typing for prior and some current donors. Interestingly, AMR among the repeat mismatch cohort was observed only in the participant where A24 was recrossed (participant 3), although the AMR may have been due to class II DSA in that case. However, the strongest rebound (neat MFI and titer) was observed in the participant where the repeat mismatch was an HLA-DQ antigen. Given the limited sample in this cohort, the significant rebound, and the immunogenicity of HLA-DQ antigens,^29^ recrossing HLA sensitization needs careful consideration of risk/benefit and posttransplant management.

We acknowledge several limitations of this study. Prospective determination of treatment strategies and clinical monitoring was only provided for the first year posttransplant, leading to heterogenous treatment and monitoring beyond that period, although most patients continued to be maintained with standard-of-care immunosuppression and monitoring. The small size of this cohort also limits our ability to provide anything but descriptive analyses. Importantly, only 2 participants had biopsies beyond the first year posttransplant; therefore, we cannot comment on the presence of subclinical chronic AMR. Complete biopsy timing and scoring data are available in **Table S1** (**SDC,** http://links.lww.com/TXD/A733). The inability to ascertain antigen expression within the graft limits our ability to conclude whether tolerogenic mechanisms are in play when DSA persists or whether simply no targets for these antibodies are accessible within the graft. Furthermore, our interpretation of DSA strength is constrained by the current limitations of the prevalent techniques for evaluating the presence of such antibodies. Although single-antigen bead approaches with titer dilutions represent a proxy for combined antibody concentration and antigen-binding strength, they are unable to reliably inform us of the functional effects of the antibody in mediating direct and cell-mediated damage.

This case series demonstrates that imlifidase-enabled HLA incompatible kidney transplants have good 5-y survival and graft function (comparable with other desensitization approaches),^25^ with late acute AMR appearing to be rare. DSA rebound is common but with heterogenous long-term trajectories. Variable DSA rebound patterns were observed here, and the sample size limits our ability to make predictive inferences about the specific risks for this. The full implications of differential long-term DSA trajectories—especially on late and chronic AMR—require further clinical and mechanistic study. Guideline committees should consider recommending DSA monitoring beyond 1 y, given relevant non-imlifidase data and the likely need for future retrospective analyses specific to imlifidase. Clinical management and biopsy decisions for patients treated with imlifidase should be driven by graft function or the combination of graft function and DSA levels, and probably not by DSA levels alone.

## ACKNOWLEDGMENTS

The authors wish to thank the participants of the Writing for Scientific Publication course hosted by the Clinical and Translational Science Institute at the New York University Grossman School of Medicine who provided valuable feedback on early drafts of this article, in particular Arthur H. Fierman, MD; Mark D. Schwartz, MD; Luke Bonanni; and Sommer E. Gentry, PhD.

## Supplementary Material



## References

[1] Fidler SJ Irish AB Lim W. Pre-transplant donor specific anti-HLA antibody is associated with antibody-mediated rejection, progressive graft dysfunction and patient death. Transpl Immunol. 2013;28:148–153. 23665534 10.1016/j.trim.2013.05.001

[2] Hönger G Hopfer H Arnold ML. Pretransplant IgG subclasses of donor-specific human leukocyte antigen antibodies and development of antibody-mediated rejection. Transplantation. 2011;92:41–47. 21637140 10.1097/TP.0b013e31821cdf0d

[3] Riethmüller S Ferrari-Lacraz S Müller MK. Donor-specific antibody levels and three generations of crossmatches to predict antibody-mediated rejection in kidney transplantation. Transplantation. 2010;90:160–167. 20658760 10.1097/tp.0b013e3181e36e08

[4] Choi AY Manook M Olaso D. Emerging new approaches in desensitization: targeted therapies for HLA sensitization. Front Immunol. 2021;12:694763. 34177960 10.3389/fimmu.2021.694763 PMC8226120

[5] Jackson KR Motter JD Kernodle A. How do highly sensitized patients get kidney transplants in the United States? Trends over the last decade. Am J Transplant. 2020;20:2101–2112. 32065704 10.1111/ajt.15825 PMC8717833

[6] Jackson KR Holscher C Motter JD. Posttransplant outcomes for cPRA-100% recipients under the new kidney allocation system. Transplantation. 2020;104:1456–1461. 31577673 10.1097/TP.0000000000002989 PMC7103562

[7] Maldonado A Sjöholm K Lee J. Beyond CPRA: identifying sensitized kidney candidates with markedly low access to deceased donor transplantation by granular CPRA and blood type. OBM Transplant. 2021;5:1–15.

[8] Schinstock CA Smith BH Montgomery RA. Managing highly sensitized renal transplant candidates in the era of kidney paired donation and the new kidney allocation system: is there still a role for desensitization? Clin Transplant. 2019;33:e13751. 31769104 10.1111/ctr.13751

[9] Orandi BJ Luo X Massie AB. Survival benefit with kidney transplants from HLA-incompatible live donors. N Engl J Med. 2016;374:940–950. 26962729 10.1056/NEJMoa1508380 PMC4841939

[10] Ge S Chu M Choi J. Imlifidase inhibits HLA antibody-mediated NK cell activation and antibody-dependent cell-mediated cytotoxicity (ADCC) in vitro. Transplantation. 2020;104:1574–1579. 32732834 10.1097/TP.0000000000003023

[11] Huang E Maldonado AQ Kjellman C. Imlifidase for the treatment of anti-HLA antibody-mediated processes in kidney transplantation. Am J Transplant. 2022;22:691–697. 34467625 10.1111/ajt.16828 PMC9293130

[12] Järnum S Bockermann R Runström A. The bacterial enzyme IdeS cleaves the IgG-type of B cell receptor (BCR), abolishes BCR-mediated cell signaling, and inhibits memory B cell activation. J Immunol. 2015;195:5592–5601. 26553074 10.4049/jimmunol.1501929 PMC4671093

[13] Jordan SC Lorant T Choi J. IgG endopeptidase in highly sensitized patients undergoing transplantation. N Engl J Med. 2017;377:442–453. 28767349 10.1056/NEJMoa1612567

[14] Jordan SC Legendre C Desai NM. Imlifidase desensitization in crossmatch-positive, highly sensitized kidney transplant recipients: results of an international phase 2 trial (Highdes). Transplantation. 2021;105:1808–1817. 33093408 10.1097/TP.0000000000003496 PMC8294837

[15] Lonze BE Tatapudi VS Weldon EP. IdeS (Imlifidase): a novel agent that cleaves human IgG and permits successful kidney transplantation across high-strength donor-specific antibody. Ann Surg. 2018;268:488–496. 30004918 10.1097/SLA.0000000000002924

[16] Kjellman C Maldonado AQ Sjöholm K. Outcomes at 3 years posttransplant in imlifidase‐desensitized kidney transplant patients. Am J Transplant. 2021;21:3907–3918. 34236770 10.1111/ajt.16754 PMC9290474

[17] European Medicines Agency. EMA. Idefirix. Available at https://www.ema.europa.eu/en/medicines/human/EPAR/idefirix. Accessed October 23, 2023

[18] Halleck F Böhmig GA Couzi L. A randomized trial comparing imlifidase to plasmapheresis in kidney transplant recipients with antibody‐mediated rejection. Clin Transplant. 2024;38:e15383. 39023092 10.1111/ctr.15383

[19] Levey AS Stevens LA Schmid CH; CKD-EPI (Chronic Kidney Disease Epidemiology Collaboration). A new equation to estimate glomerular filtration rate. Ann Intern Med. 2009;150:604–612. 19414839 10.7326/0003-4819-150-9-200905050-00006 PMC2763564

[20] Beadle J Papadaki A Toulza F. Application of the Banff human organ transplant panel to kidney transplant biopsies with features suspicious for antibody-mediated rejection. Kidney Int. 2023;104:526–541. 37172690 10.1016/j.kint.2023.04.015

[21] Tambur AR Wiebe C. HLA diagnostics: evaluating DSA strength by titration. Transplantation. 2018;102:S23–S30. 29266059 10.1097/TP.0000000000001817

[22] Schiavo T Montagner J Keitel E. The Halifax flow crossmatch protocol results according to the class and MFI of the DSA. Braz J Transplant. 2023;26:e1223.

[23] Zeevi A Lunz J. HLA antibody profiling in thoracic transplantation undergoing desensitization therapy. Curr Opin Organ Transplant. 2012;17:416–422. 22790076 10.1097/MOT.0b013e328355f1ab

[24] Duquesnoy RJ Marrari M. Detection of antibodies against HLA-C epitopes in patients with rejected kidney transplants. Transpl Immunol. 2011;24:164–171. 21185937 10.1016/j.trim.2010.12.003

[25] Marfo K Lu A Ling M. Desensitization protocols and their outcome. Clin J Am Soc Nephrol. 2011;6:922–936. 21441131 10.2215/CJN.08140910

[26] Salvadori M Bertoni E. Impact of donor-specific antibodies on the outcomes of kidney graft: pathophysiology, clinical, therapy. World J Transplant. 2014;4:1–17. 24669363 10.5500/wjt.v4.i1.1 PMC3964192

[27] Lobashevsky AL Higgins NG Rosner KM. Analysis of anti-HLA antibodies in sensitized kidney transplant candidates subjected to desensitization with intravenous immunoglobulin and rituximab. Transplantation. 2013;96:182–190. 23778648 10.1097/TP.0b013e3182962c84

[28] Shaffer D Feurer ID Crowe D. Early and sustained reduction in donor-specific antibodies in desensitized living donor kidney transplant recipients: a 3-year prospective study. Transplant Direct. 2016;2:e62. 27500255 10.1097/TXD.0000000000000570 PMC4946491

[29] DeVos JM Gaber AO Knight RJ. Donor-specific HLA-DQ antibodies may contribute to poor graft outcome after renal transplantation. Kidney Int. 2012;82:598–604. 22622504 10.1038/ki.2012.190

