# Supplementary material for: Clinical Outcomes and Donor-specific Antibody Rebound 5 y After Kidney Transplant Enabled by Imlifidase Desensitization
Source: Transplant Direct. 2025 Jan 9;11(2):e1752. doi: 10.1097/TXD.0000000000001752 (PMC11723687; doi:10.1097/TXD.0000000000001752)
Supplement: Supplementary file 1 [file txd-11-e1752-s001.pdf]

Supplementary Materials

Supplement to: IS Jaffe, VS Tatapudi, EP Weldon, et al. Clinical Outcomes and Donor-Specific Antibody Rebound Five Years After Kidney Transplant Enabled by Imlifidase Desensitization

Table of Contents:

Abbreviations for Supplement **page 2**

Clinical Summary of Participants **page 3**
Participant 1 page 3
Participant 2 page 3
Participant 3 page 4
Participant 4 page 5
Participant 5 page 6
Participant 6 page 6
Participant 7 page 7
Participant 8 page 7

Supplementary Figures **page 9**
Figure S1: Clinical Outcomes and DSA Levels with Titrations for Participant 1 page 9
Figure S2: Participant 1 Third-Party Antibodies page 10
Figure S3: Clinical Outcomes and DSA Levels with Titrations for Participant 2 page 11
Figure S4: Participant 2 Third-Party Antibodies page 12
Figure S5: Clinical Outcomes and DSA Levels with Titrations for Participant 3 page 13
Figure S6: Participant 3 Third-Party Antibodies page 14
Figure S7: Clinical Outcomes and DSA Levels with Titrations for Participant 4 page 15
Figure S8: Participant 4 Third-Party Antibodies page 16
Figure S9: Clinical Outcomes and DSA Levels with Titrations for Participant 5 page 17
Figure S10: Participant 5 Third-Party Antibodies page 18
Figure S11: Clinical Outcomes and DSA Levels with Titrations for Participant 6 page 19
Figure S12: Participant 6 Third-Party Antibodies page 20
Figure S13: Clinical Outcomes and DSA Levels with Titrations for Participant 7 page 21
Figure S14: Participant 7 Third-Party Antibodies page 22
Figure S15: Clinical Outcomes and DSA Levels with Titrations for Participant 8 page 23
Figure S16: Participant 8 Third-Party Antibodies page 24
Figure S17: eGFR Pre- and Post-Transplant page 25
Figure S18: Aggregates of Dilutional Titrations for Third-Party Antibody Strength page 26

Supplementary Tables **page 27**
Table S1: Renal Biopsies and Banff Scoring page 27

Abbreviations for Supplement

AMR, antibody mediated rejection

CMV, cytomegalovirus

CNI, calcineurin inhibitor

cPRA, calculated panel reactive antibody

DBD, donation after brain death

DNR, do not resuscitate

DSA, donor-specific antibody

ESKD, end-stage kidney disease

FCXM, flow cytometry crossmatch

HLA, human leukocyte antigen

IVIG, intravenous immunoglobulin

MFI, mean fluorescence intensity

POD, post-operative day

SAB, single antigen bead.

Clinical Summary of Participants

Participant 1

Participant 1 was a 64-year-old man with ESKD of unknown etiology and a cPRA of 100% who had been on dialysis for 9.1 years after his prior kidney transplant had failed (Figure S1). He received a DBD donor offer that was HLA-incompatible due to the presence of a strong HLA-DPB1*17:01 antibody that had an undiluted MFI of 21,427 (titer 1:64). After desensitization with two doses of imlifidase, the DSA decreased to 2,106 MFI. After transplant, the DSA rebounded slowly, reaching an undiluted peak MFI value of 11,486 at POD28 (titer <1:16), which was predictive of a positive FCXM. AMR was specifically excluded in a biopsy on POD 12 and again in a biopsy at 6 months post-transplant. Third-party antibodies also partially rebounded but remained below baseline (Figure S2). The participant developed low-level BK viremia 3 months post-transplant and CMV viremia 11 months post-transplant, which was treated with valganciclovir. The participant also had two hospitalizations for infection. The second of these was an influenza infection complicated by secondary bacterial pneumonia that resulted in sepsis leading to cardiac arrest and death.

Participant 2

Participant 2 was a 45-year-old woman with ESKD due to polycystic kidney disease and a cPRA of 100% due to repeated blood transfusions secondary to chronic anemia (Figure S3). She had been on dialysis for 23.3 years. She received a DBD organ offer that was HLA-incompatible due to the presence of both Class I and II DSAs (A1: 23,909 MFI/titer 1:64; B27: 20,009/<1:16; B38: 5,973/<1:16; Cw9: 22,053/1:16; Cw12: 19,714/1:16; DQ6: 5,122/<1:16). After imlifidase desensitization, A1 DSA decreased to 3,228 MFI, B27 to 543, Cw9 to 1,769, C*12 to 1,296, and DQ6 to 118 (all virtually negative at 1:16). Despite minimal DSA

levels on PODs 1 and 7, all not predictive of a positive FCXM (max 4,827 MFI), the participant had delayed graft function and a biopsy performed on POD9 demonstrated AMR. By then there had been a precipitous increase in her rebounding DSAs (A1: 23,965 neat MFI, B8: 5,125 MFI, cw9: 11,560 MFI, cw12: 9,071 MFI, DQ6: 8,203 MFI). She was treated with plasmapheresis, eculizumab, and bortezomib, as well as functional splenectomy via splenic artery embolization, with resulting establishment of graft function. By five years post-transplant, DSA levels rebounded slightly and were predictive of a positive FCXM only due to the cumulative MFI value (A1: 4,375 MFI; B27: 3,531; B38: 701; Cw9: 3,998; Cw12: 4,563; DQ6: 1,896) (all virtually negative at 1:16). However, AMR never recurred, and no immunosuppression beyond standard triple therapy was required. Third-party antibody levels similarly rebounded only slightly (Figure S4). The participant had no infection-related hospitalizations but did have a one-week period of CMV viremia that resolved with valgancyclovir.

Participant 3

Participant 3 was a 31-year-old man with ESKD of unknown etiology and a cPRA of 99% who had been on dialysis for 7.3 years after his prior kidney transplant had failed (Figure S5). He received a DBD offer that was HLA-incompatible due to the presence of DSA against A24 (6,303 MFI; titer <1:16) and DQ4 (18,561; titer 1:32). After imlifidase desensitization, both DSAs decreased to <100 MFI. Although the A24 DSA never rebounded, despite being a repeat mismatch, the DQ4 DSA began to rebound at POD 7 and reached a peak of 18,043 MFI (titer >1:128) by POD24. Definitive AMR was demonstrated on a biopsy on POD26 and was treated with plasmapheresis, with subsequent establishment of partial graft function. The participant's allograft demonstrated significant donor-derived vascular disease and fibrosis that had not been fully appreciated on pre-transplant biopsy, and the participant was relisted for transplant after 36 months (no re-transplant had occurred, nor had the participant required maintenance dialysis

through five years post-transplant). During the five years, baseline graft function remained unchanged, without recurrence of AMR, despite anti-DQ4 DSA levels rebounding to greater than undiluted baseline and with a higher titer (23,949 MFI/>1:128 at five years vs. 18,874 MFI/1:32 at baseline). Third-party antibodies mirrored the DQ4 DSA, rebounding to a level greater than baseline (Figure S6). The participant had four infection-related hospitalizations and a brief period of CMV viremia that resolved with valgancyclovir.

Participant 4

Participant 4 was a 40-year-old man with ESKD due to diabetes mellitus and a cPRA of 100% who had been on dialysis for 4.5 years after his prior kidney transplant had failed (Figure S7). He received a DBD organ offer which was incompatible due to the presence of strong DSA against DPB1*04:01/DPA1*01:03 (neat 21,493 MFI, titer 1:32). After imlifidase desensitization, HLA-DP DSA was <1,800 MFI (negative at 1:16). Within the first week, the participant had delayed graft function and rebound of HLA-DP DSA to levels predictive of a positive FCXM (6,716 MFI). At POD28, the DP DSA increased to 14,323 MFI, but demonstrated a significant decrease in titer (<1:16) as compared to the baseline. He was treated for biopsy-proven AMR with plasma exchange starting on POD6, with recovery of graft function. AMR never recurred despite DSA levels persistently predictive of positive FCXMs. DSA levels remained below baseline through year five, as did third-party HLA antibodies (Figure S8). The participant developed calcineurin inhibitor toxicity three months post-transplant and was switched to belatacept for CNI elimination. The participant had four infection-related hospitalizations.

Participant 5

Participant 5 was a 31-year-old woman with ESKD due to systemic lupus erythematosus and a cPRA of 98% who had been on dialysis for 7.4 years after her most recent kidney transplant had failed (Figure S9). She received a DBD donor offer that was HLA-incompatible due to the presence of a strong DQ7 DSA (neat MFI 22,193; titer 1:64). After imlifidase desensitization, the DSA MFI decreased to 3,668 (negative at 1:16). During the first month, her DSA levels rebounded slightly (maximum median MFI 3,668 at 24 hours), maintained a low titer (negative at 1:16), and was never predictive of a positive FCXM. By five years post-transplant, DSA levels had rebounded to a median 11,760 MFI, predictive of a positive FCXM, but classified as low AMR risk on dilutional titer testing (titer <1:16). The participant never developed AMR. Third-party antibodies similarly rebounded but remained below baseline levels (Figure S10). The participant had two infection-related hospitalizations. Notably, the participant uniquely had persistently low total IgG levels, necessitating multiple IVIG infusions for hypogammaglobulinemia during the first-year post-transplant.

Participant 6

Participant 6 was a 33-year-old man with ESKD due to congenital reflux nephropathy and a cPRA of 99% who had been on dialysis for 6.6 years after his prior kidney transplant had failed (Figure S11). He received a living donor organ transplant which was HLA-incompatible due to the presence of an HLA-DR12 antibody with an undiluted MFI of 8,680 (titer <1:16) which was a repeat mismatch from his previous transplant. After desensitization with imlifidase, the DR12 DSA decreased to 208 MFI and at 5 years the DSA MFI was 441 (negative at 1:16). The participant never developed AMR. Interestingly, although the participants' DSA did not rebound, third-party antibodies rebounded significantly, with

some Class I third-party antibodies exceeding pre-transplant baseline (Figure S12). The participant had five infection-related hospitalizations, including one related to CMV viremia causing pneumonitis, which resolved with valgancyclovir treatment.

Participant 7

Participant 7 was a 42-year-old woman with ESKD due to hypertension and a cPRA of 100% who had been on dialysis for 0.8 year after her prior kidney transplant had failed (Figure S13). She received a living donor organ offer that was HLA-incompatible due to the presence of a strong DQ5 DSA which was also a repeat mismatch (24,056; titer >1:128), and a strong DQ6 (23,220; titer >1:128). After imlifidase desensitization, DQ5 was reduced to 3,004 MFI, and DQ6 to 2,309 MFI. Both Class II DSAs rebounded by POD7 to levels predictive of a positive FCXM and with high-risk dilutional titers (DQ5: 17,925 MFI/1:64; DQ6: 14,732/1:32). Both DQ5 and DQ6 DSAs continue to increase through at least POD28 where they reached a peak expression and titer (DQ5: 24,032 MFI/1:64; DQ6: 21,570/1:64). Interestingly, and despite the repeat mismatch at DQ5, DSAs levels decreased significantly between day 28 and five-years post-transplant. While the total DSA MFI level at five years post-transplant (7,616 MFI) were predictive of a positive FCXM, they no longer represented a high-risk antibody since their titer changed from >8000 MFI at 1:64 to <2000 MFI at 1:16 dilution. A similar pattern of partial rebound at day 28 followed by quiescence at five years was seen for third-party antibodies (Figure S14). The participant never developed AMR and had no significant infections.

Participant 8

Participant 8 was a 39-year-old man with ESKD of unknown etiology and a cPRA of 96% who had been on dialysis for 0.7 year after his prior kidney transplant had failed (Figure 15). He received a living donor

offer that was HLA incompatible due to the presence of strong DSAs against HLA-DQ5 (23,604 MFI; titer >1:128) and HLA-DQ6 (23,301 MFI; titer >1:128). After imlifidase desensitization, the DQ5 decreased to 1,692 MFI and the DQ6 to 1,598 MFI. By POD7, both DSA rebounded in the neat serum to levels predictive of a positive FCXM (DQ5 7,708 MFI; DQ6 7,619 MFI), however their titer dropped to <1:16. DSAs remained at levels predictive of a positive FCXM through five years post-transplant, although with a titer level <1:16 and without incidence of AMR. Third-party antibodies followed a similar trajectory (Figure S16). The participant had no significant infectious complications.

Supplementary Figures

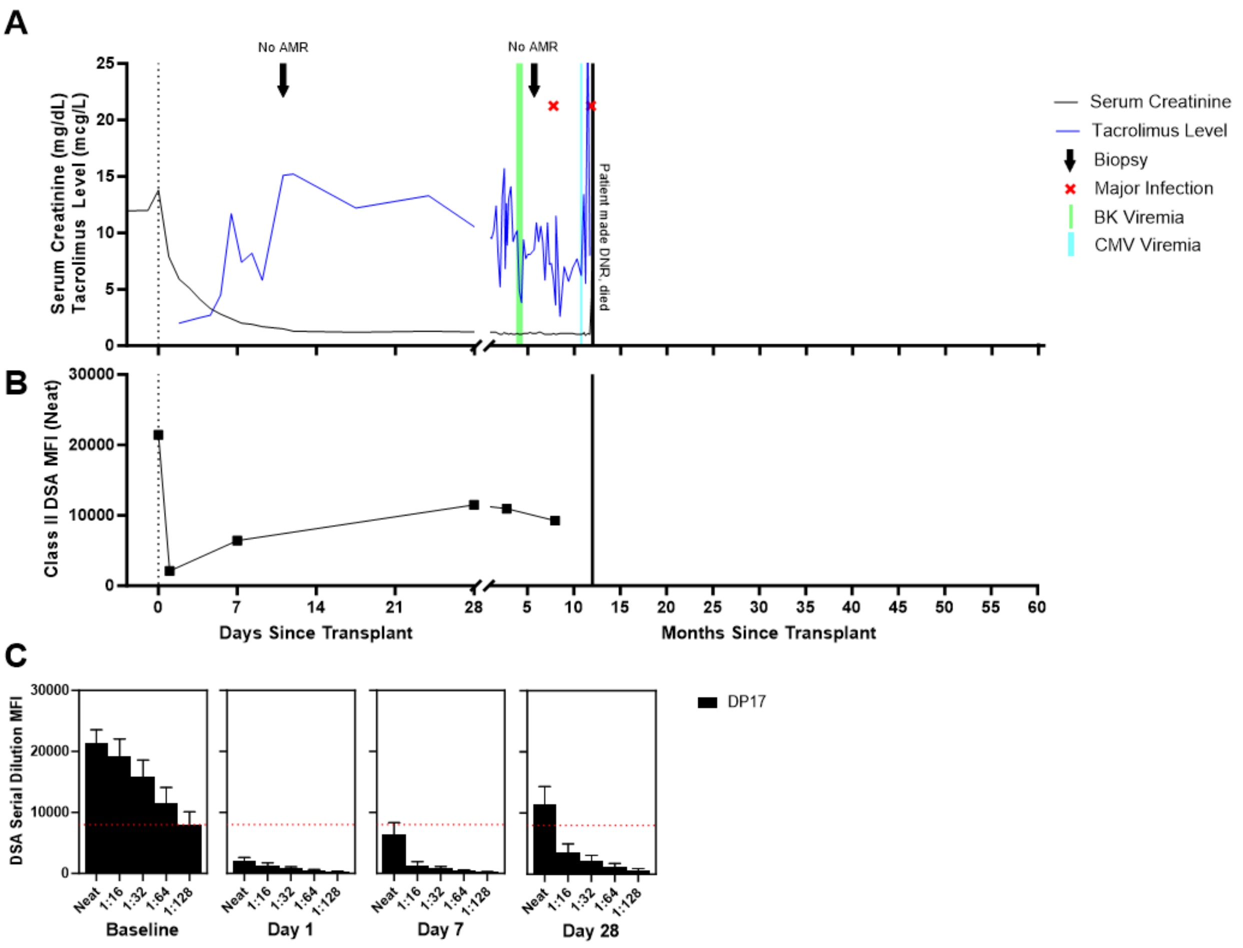

Figure S1: Clinical outcomes and DSA levels with titrations for Participant 1. A) shows the participant's serum creatinine over time, biopsy results, and immunomodulating treatments. B) shows DSA MFIs for each SAB. Panels A and B are presented on a common axis with the first 28 days presented in detail (left) followed by the ensuing 59 months of post-transplant follow-up (right). C) shows median MFI and standard deviation of DSA titrations performed by serial dilution, with a red dotted line at 8,000 MFI. AMR, antibody mediated rejection; DSA, donor specific antibodies; IVIG, intravenous immunoglobulin; MFI, mean fluorescence intensity; SAB, single antigen bead.

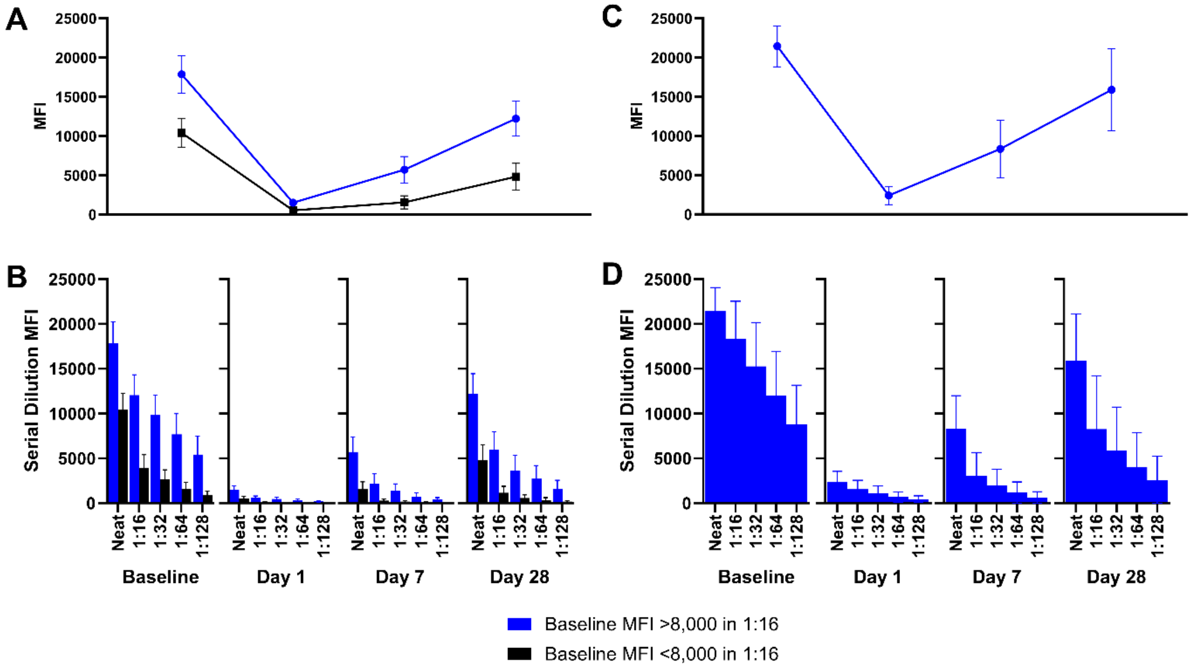

Figure S2: Participant 1 third-party antibodies with titrations A) and C) show the HLA Class I and Class II third-party antibodies stratified by antibodies with MFI >8,000 in 1:16 dilution (likely complement-fixing) at baseline. B) and D) show HLA Class I and Class II third-party antibody serial dilution titrations, stratified by the same criteria. HLA, human leukocyte antigen; MFI, mean fluorescence intensity.

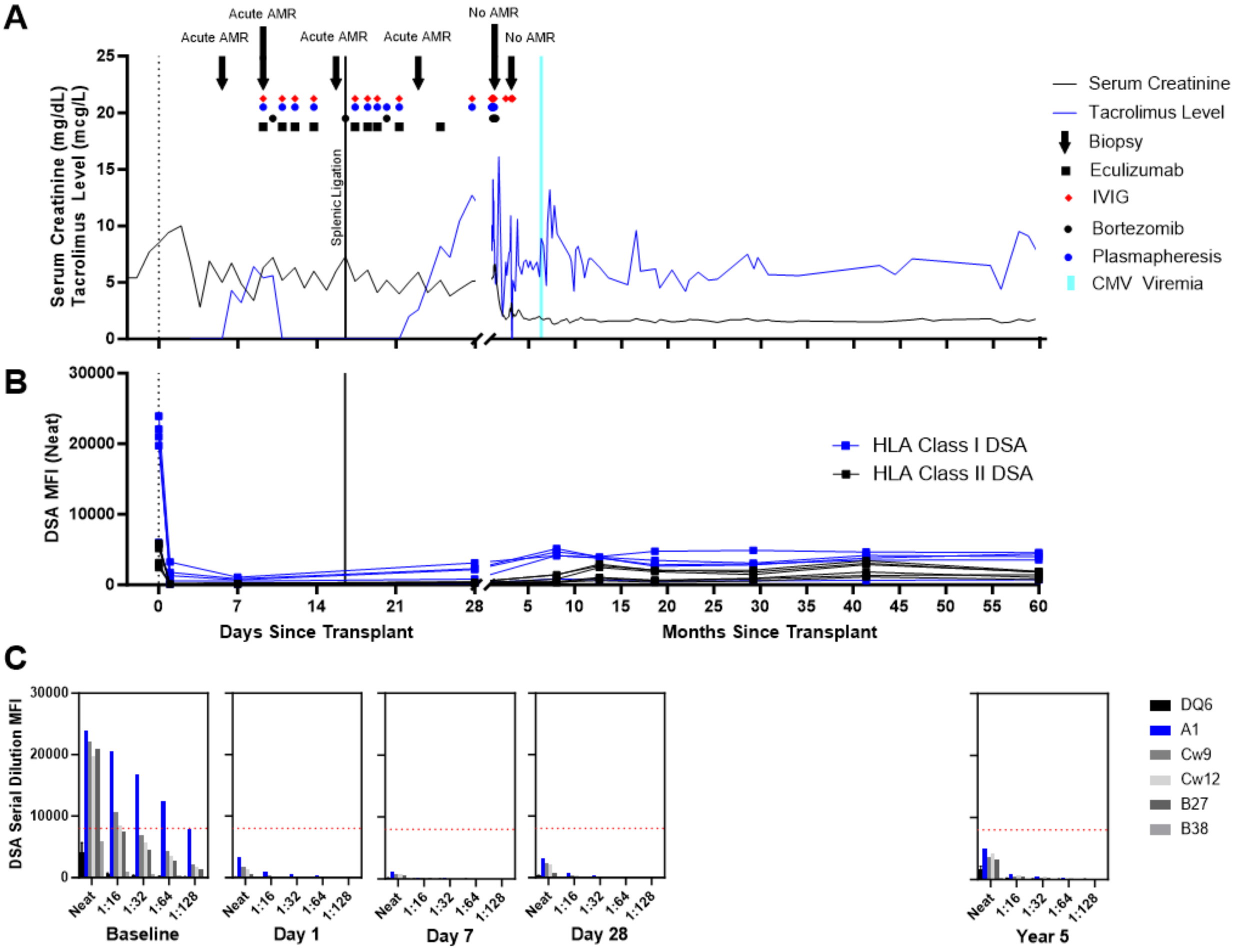

Figure S3. Clinical outcomes and DSA levels with titrations for Participant 2. A) shows the participant's serum creatinine over time, biopsy results, and immunomodulating treatments. B) shows DSA MFIs for each SAB. Panels A and B are presented on a common axis with the first 28 days presented in detail (left) followed by the ensuing 59 months of post-transplant follow-up (right). C) shows median MFI and standard deviation of DSA titrations performed by serial dilution, with a red dotted line at 8,000 MFI. AMR, antibody mediated rejection; DSA, donor specific antibodies; IVIG, intravenous immunoglobulin; MFI, mean fluorescence intensity; SAB, single antigen bead.

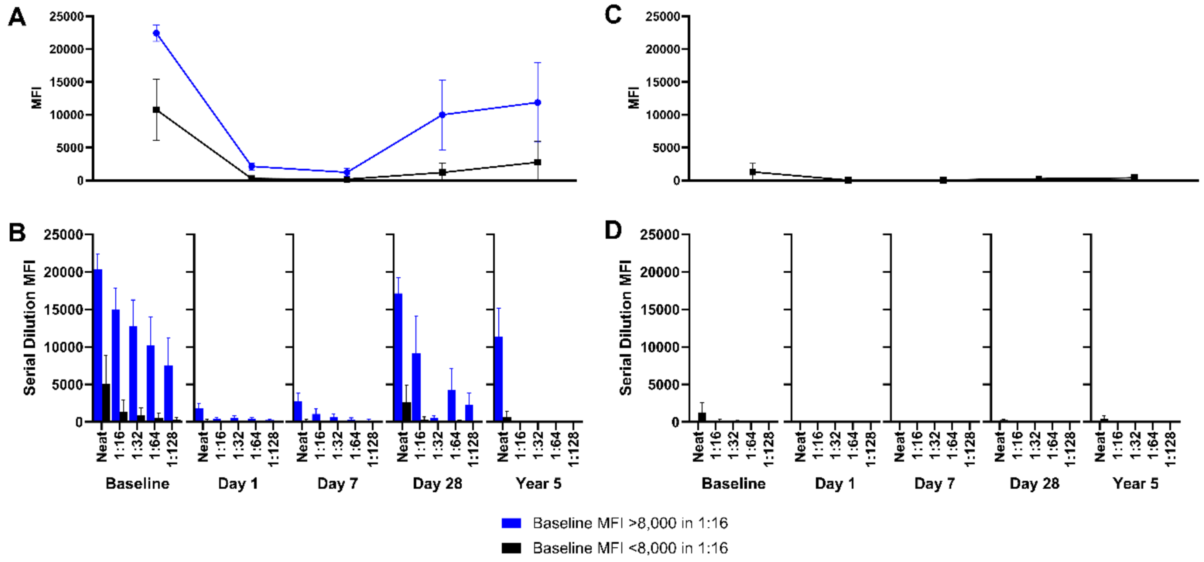

Figure S4: Participant 2 third-party antibodies with titrations. A) and C) show the HLA Class I and Class II third-party antibodies stratified by antibodies with MFI >8,000 in 1:16 dilution (likely complement-fixing) at baseline. B) and D) show HLA Class I and Class II third-party antibody serial dilution titrations, stratified by the same criteria. HLA, human leukocyte antigen; MFI, mean fluorescence intensity.

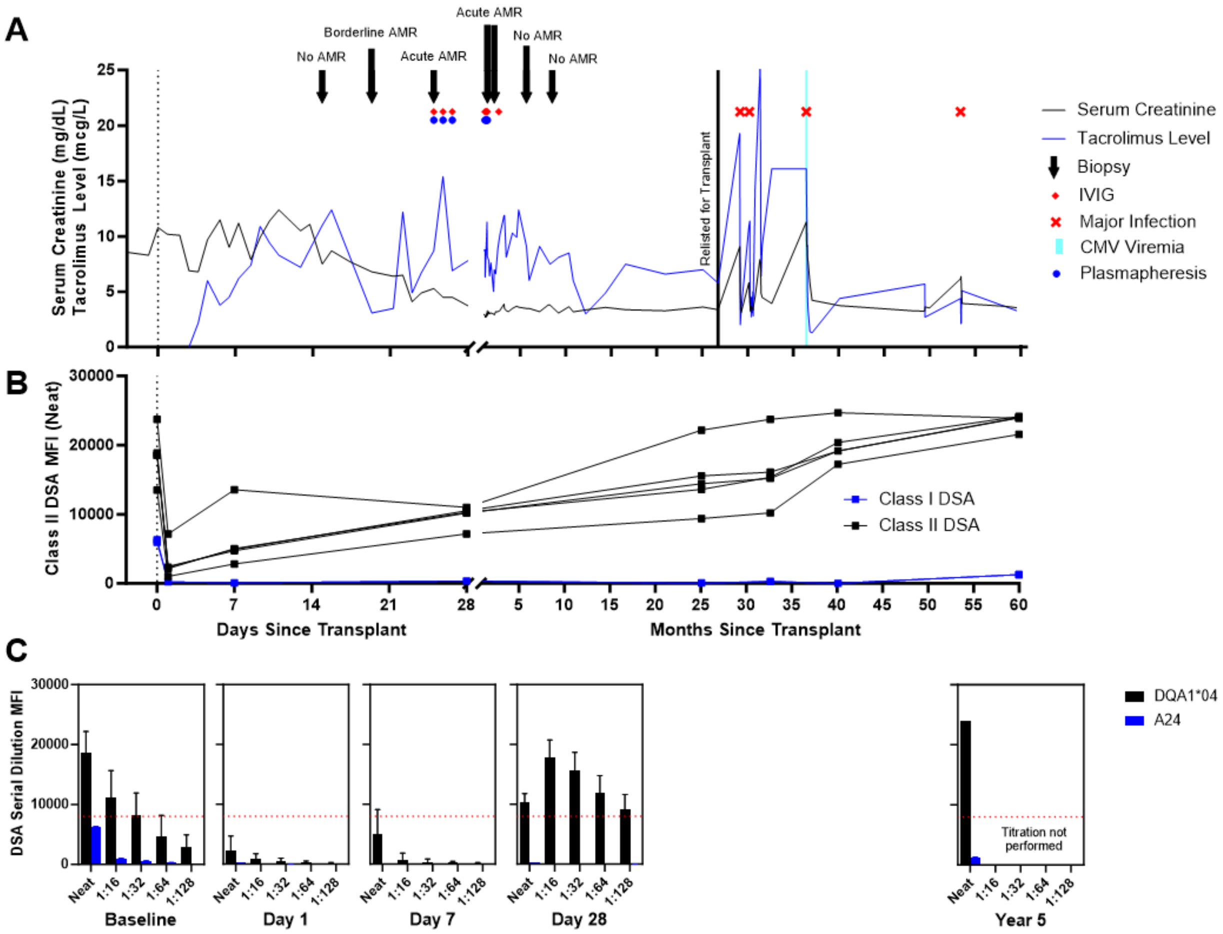

Figure S5. Clinical outcomes and DSA levels with titrations for Participant 3. A) shows the participant's serum creatinine over time, biopsy results, and immunomodulating treatments. B) shows DSA MFIs for each SAB. Panels A and B are presented on a common axis with the first 28 days presented in detail (left) followed by the ensuing 59 months of post-transplant follow-up (right). C) shows median MFI and standard deviation of DSA titrations performed by serial dilution, with a red dotted line at 8,000 MFI. AMR, antibody mediated rejection; DSA, donor specific antibodies; IVIG, intravenous immunoglobulin; MFI, mean fluorescence intensity; SAB, single antigen bead.

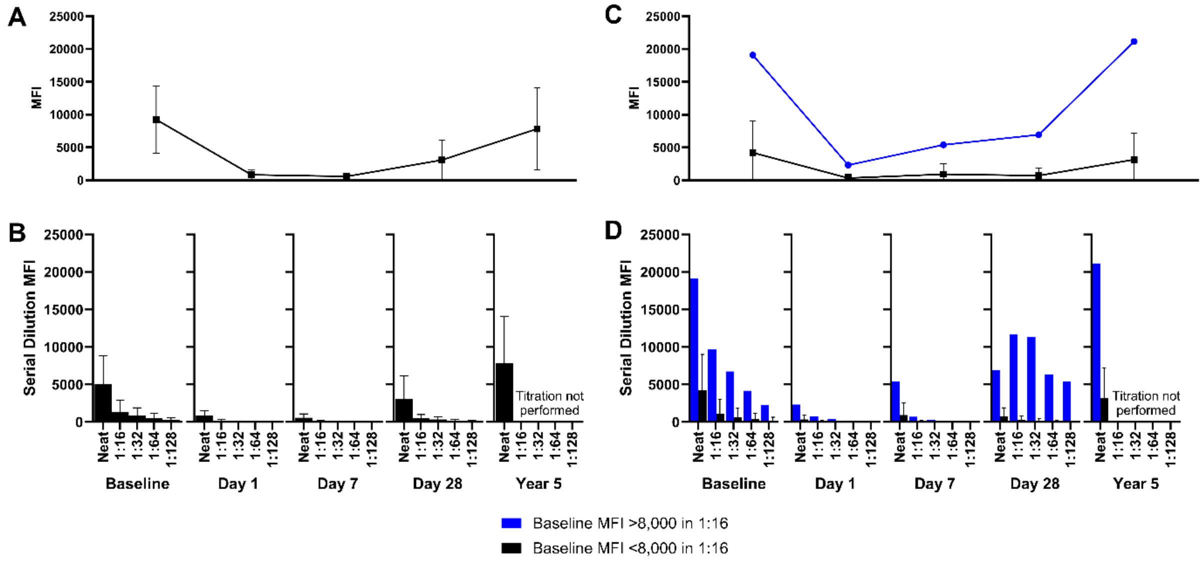

Figure S6: Participant 3 third-party antibodies with titrations. A) and C) show the HLA Class I and Class II third-party antibodies stratified by antibodies with MFI >8,000 in 1:16 dilution (likely complement-fixing) at baseline. B) and D) show HLA Class I and Class II third-party antibody serial dilution titrations, stratified by the same criteria. HLA, human leukocyte antigen; MFI, mean fluorescence intensity.

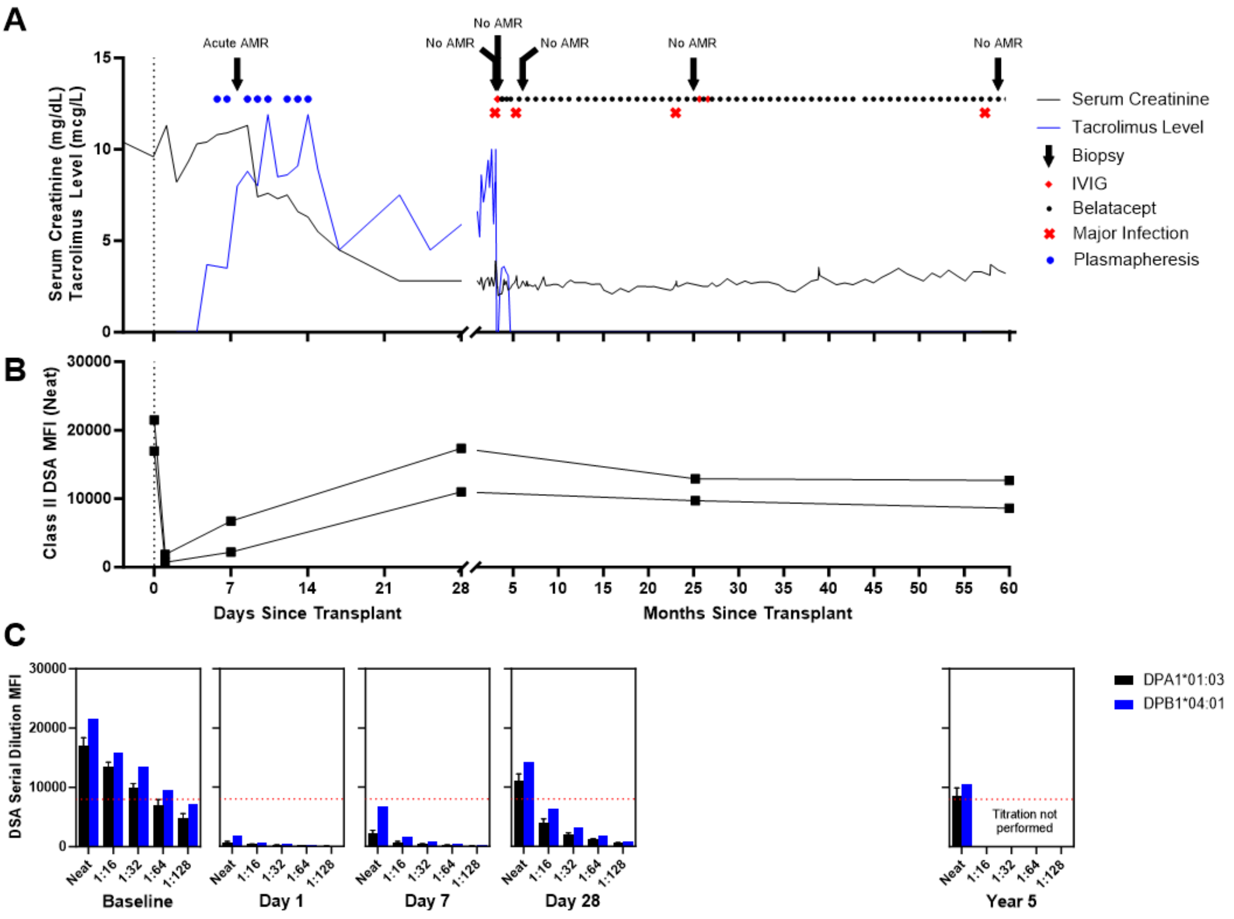

Figure S7. Clinical outcomes and DSA levels with titrations for Participant 4. A) shows the participant's serum creatinine over time, biopsy results, and immunomodulating treatments. B) shows DSA MFIs for each SAB. Panels A and B are presented on a common axis with the first 28 days presented in detail (left) followed by the ensuing 59 months of post-transplant follow-up (right). C) shows median MFI and standard deviation of DSA titrations performed by serial dilution, with a red dotted line at 8,000 MFI. AMR, antibody mediated rejection; DSA, donor specific antibodies; IVIG, intravenous immunoglobulin; MFI, mean fluorescence intensity; SAB, single antigen bead.

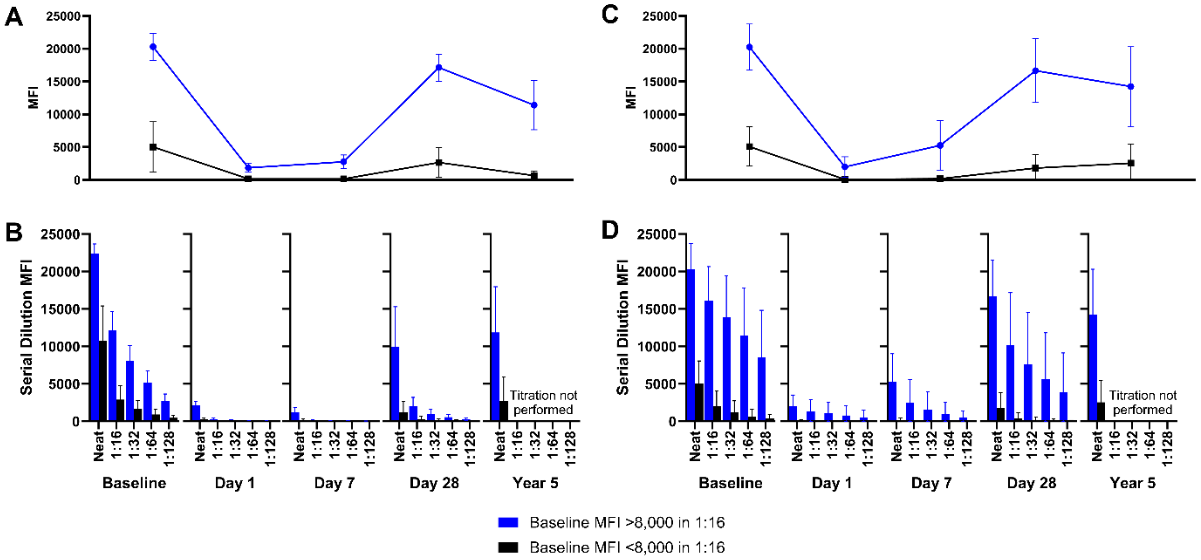

Figure S8. Participant 4 third-party antibodies with titrations. A) and C) show the HLA Class I and Class II third-party antibodies stratified by antibodies with MFI >8,000 in 1:16 dilution (likely complement-fixing) at baseline. B) and D) show HLA Class I and Class II third-party antibody serial dilution titrations, stratified by the same criteria. HLA, human leukocyte antigen; MFI, mean fluorescence intensity.

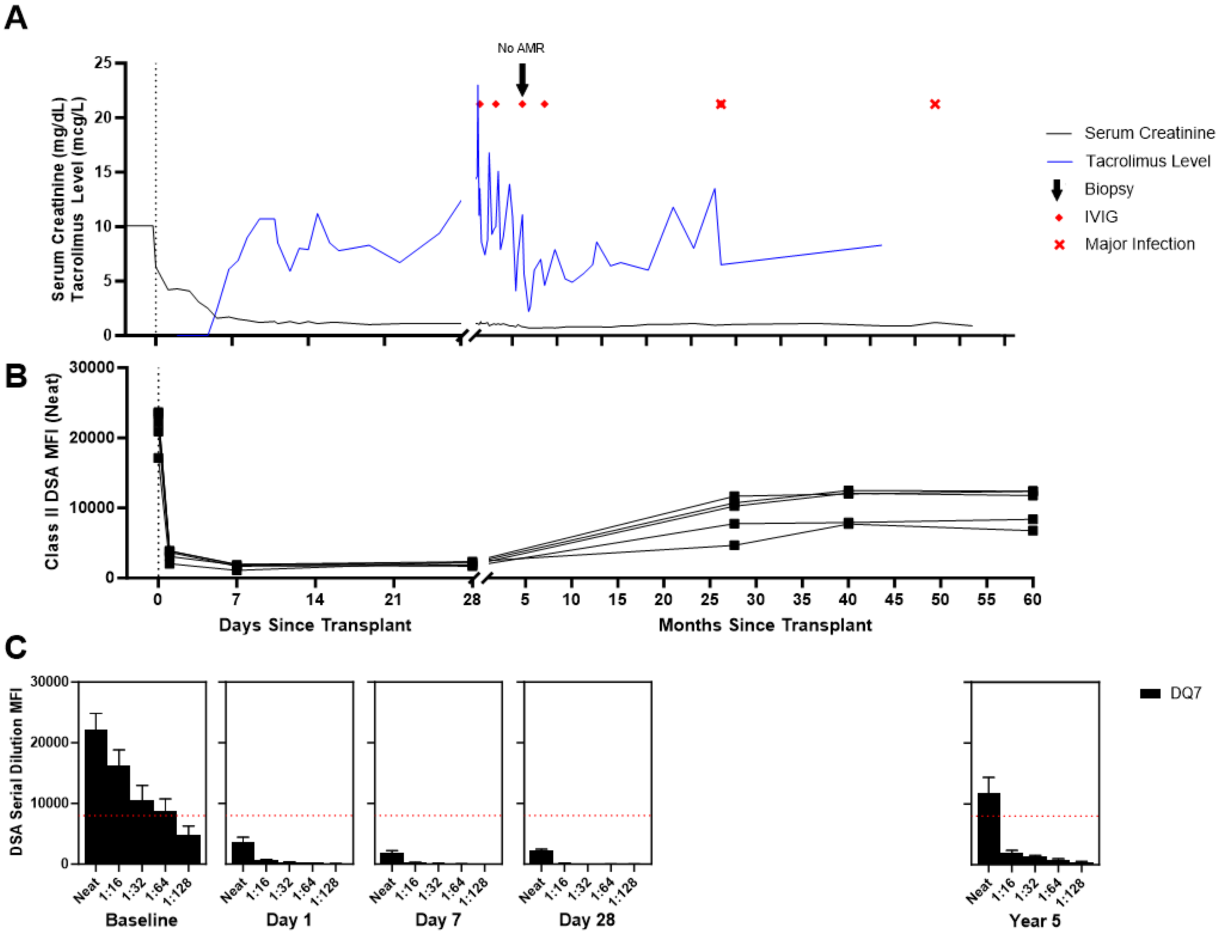

Figure S9: Clinical outcomes and DSA levels with titrations for Participant 5. A) shows the participant's serum creatinine over time, biopsy results, and immunomodulating treatments. B) shows DSA MFIs for each SAB. Panels A and B are presented on a common axis with the first 28 days presented in detail (left) followed by the ensuing 59 months of post-transplant follow-up (right). C) shows median MFI and standard deviation of DSA titrations performed by serial dilution, with a red dotted line at 8,000 MFI. AMR, antibody mediated rejection; DSA, donor specific antibodies; IVIG, intravenous immunoglobulin; MFI, mean fluorescence intensity; SAB, single antigen bead.

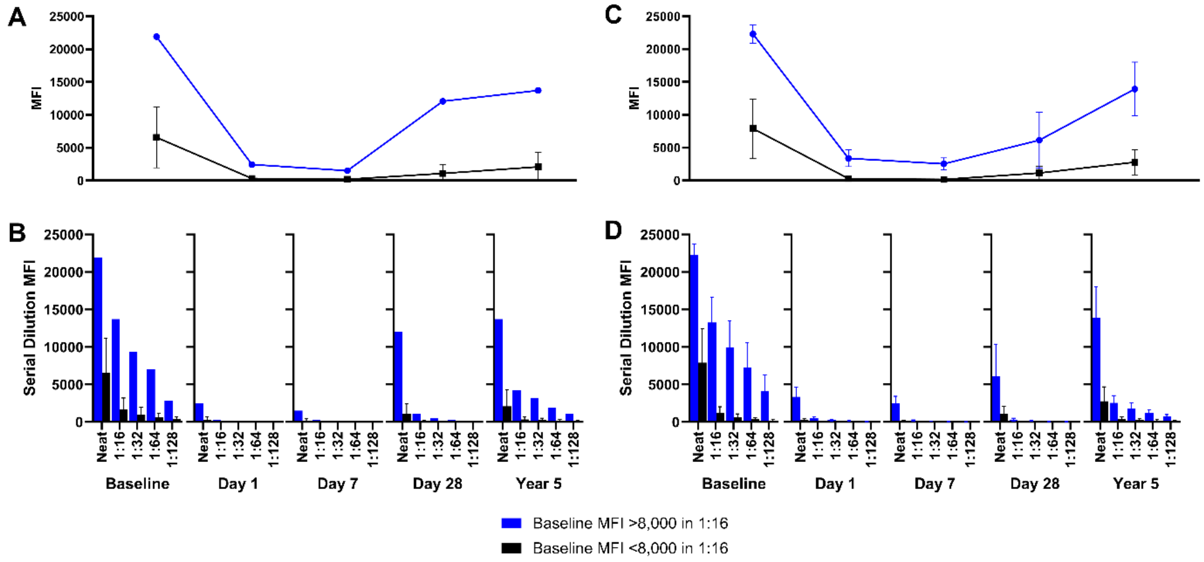

Figure S10: Participant 5 third-party antibodies with titrations. A) and C) show the HLA Class I and Class II third-party antibodies stratified by antibodies with MFI >8,000 in 1:16 dilution (likely complement-fixing) at baseline. B) and D) show HLA Class I and Class II third-party antibody serial dilution titrations, stratified by the same criteria. HLA, human leukocyte antigen; MFI, mean fluorescence intensity.

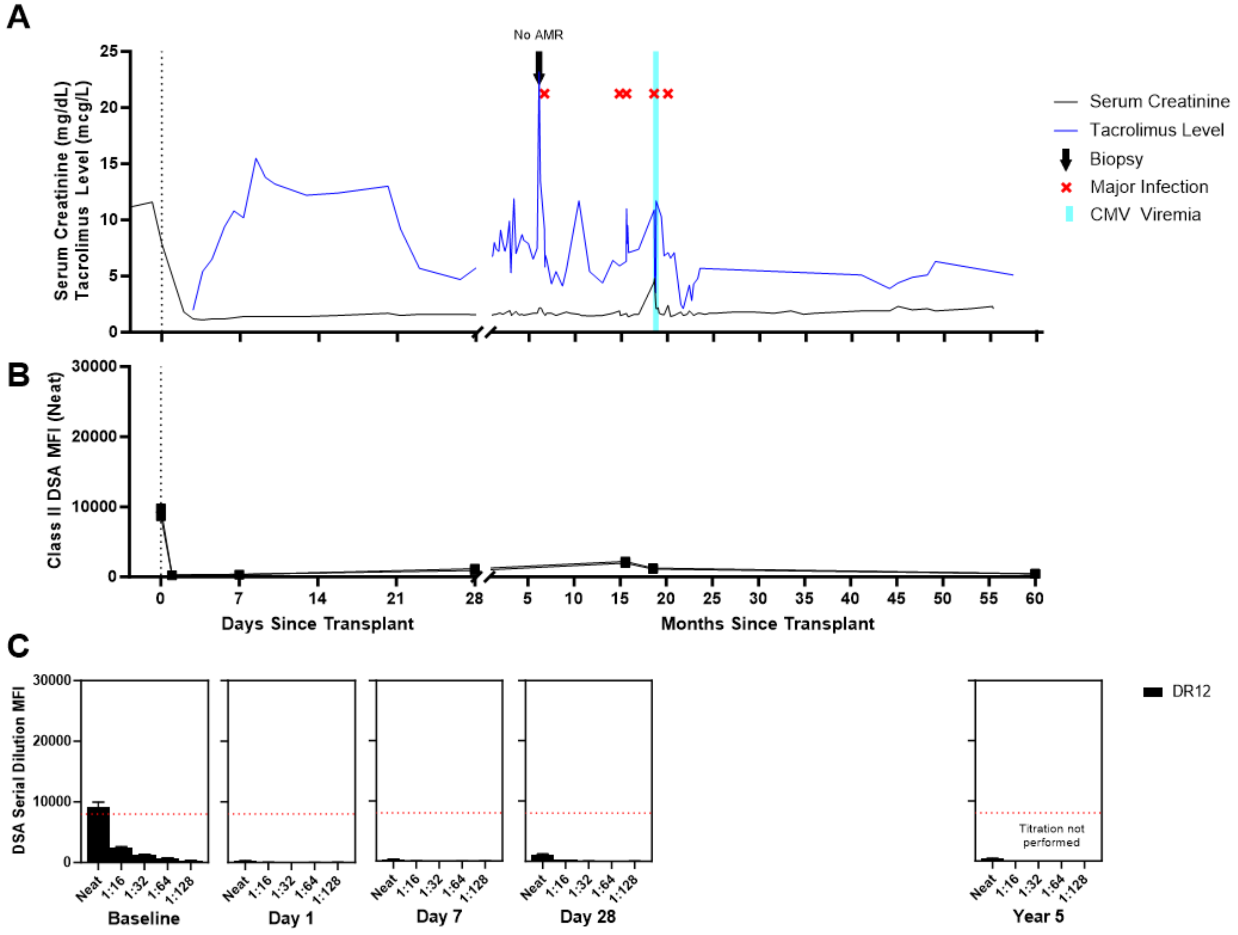

Figure S11: Clinical outcomes and DSA levels with titrations for Participant 6. A) shows the participant's serum creatinine over time, biopsy results, and immunomodulating treatments. B) shows DSA MFIs for each SAB. Panels A and B are presented on a common axis with the first 28 days presented in detail (left) followed by the ensuing 59 months of post-transplant follow-up (right). C) shows median MFI and standard deviation of DSA titrations performed by serial dilution, with a red dotted line at 8,000 MFI. AMR, antibody mediated rejection; DSA, donor specific antibodies; IVIG, intravenous immunoglobulin; MFI, mean fluorescence intensity; SAB, single antigen bead.

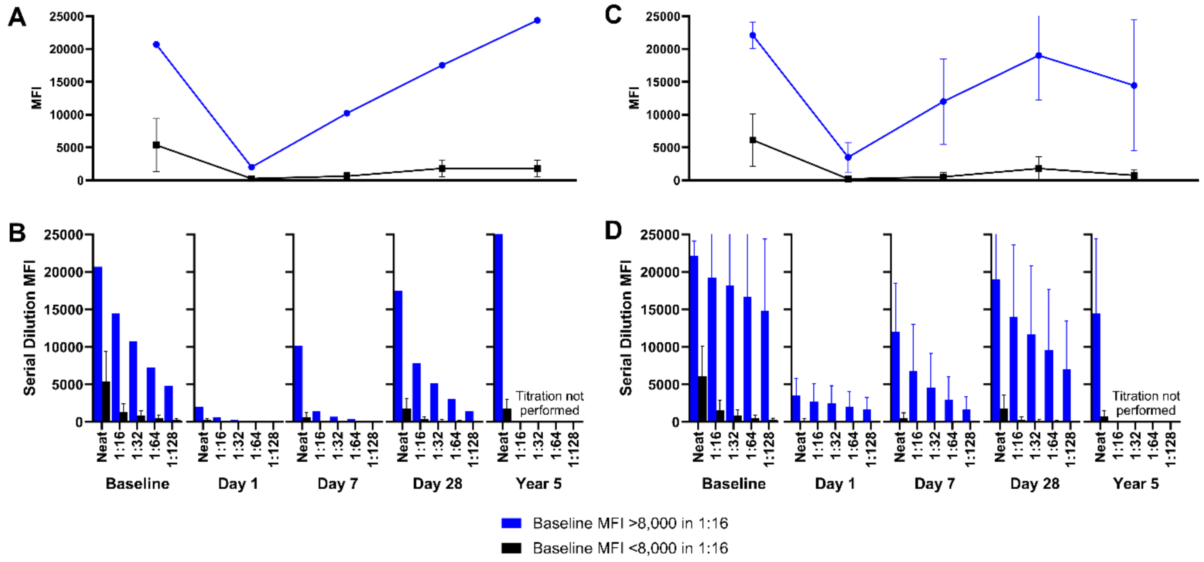

Figure S12: Participant 6 third-party antibodies with titrations. A) and C) show the HLA Class I and Class II third-party antibodies stratified by antibodies with MFI >8,000 in 1:16 dilution (likely complement-fixing) at baseline. B) and D) show HLA Class I and Class II third-party antibody serial dilution titrations, stratified by the same criteria. HLA, human leukocyte antigen; MFI, mean fluorescence intensity.

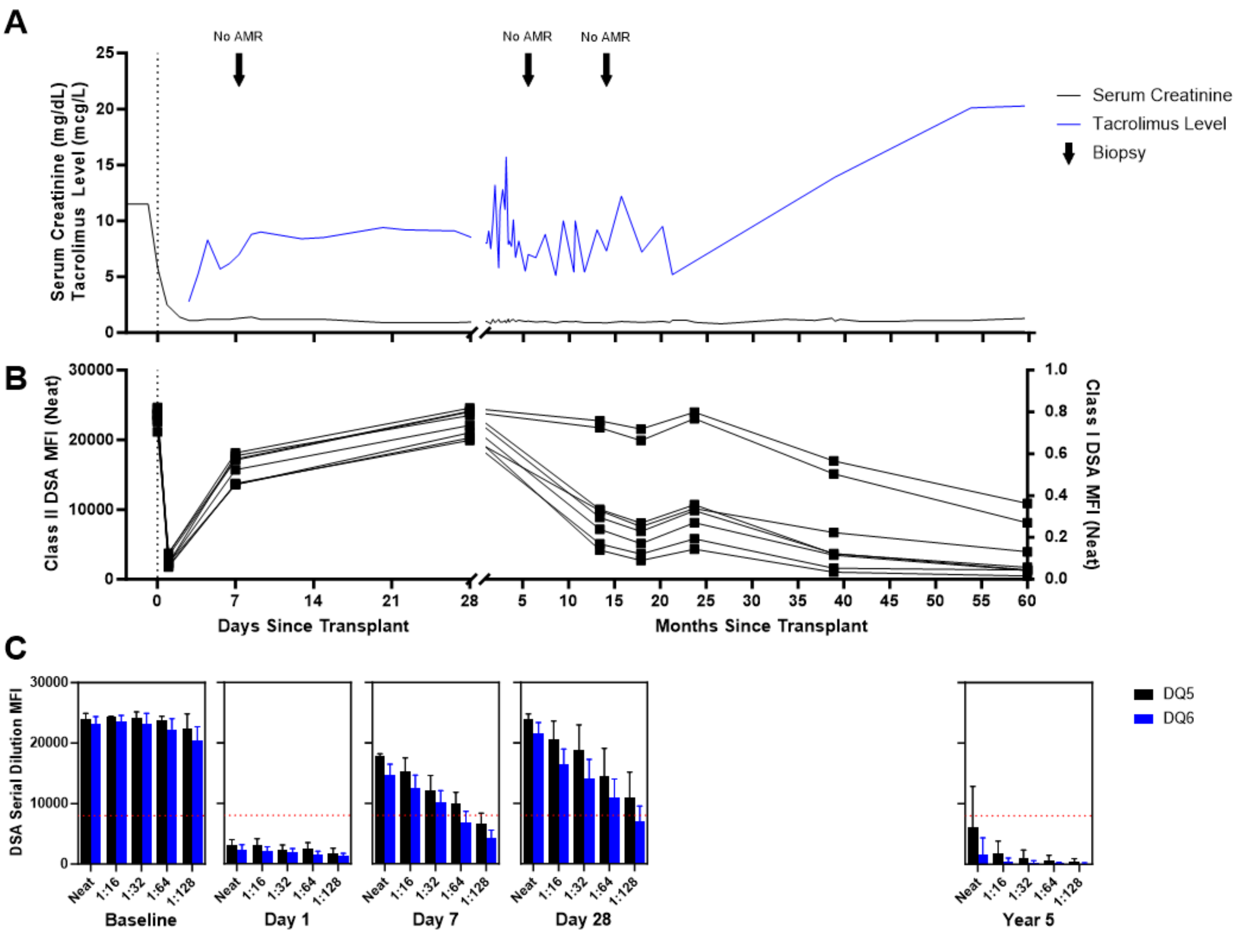

Figure S13. Clinical outcomes and DSA levels with titrations for Participant 7. A) shows the participant's serum creatinine over time, biopsy results, and immunomodulating treatments. B) shows DSA MFIs for each SAB. Panels A and B are presented on a common axis with the first 28 days presented in detail (left) followed by the ensuing 59 months of post-transplant follow-up (right). C) shows median MFI and standard deviation of DSA titrations performed by serial dilution, with a red dotted line at 8,000 MFI. AMR, antibody mediated rejection; DSA, donor specific antibodies; IVIG, intravenous immunoglobulin; MFI, mean fluorescence intensity; SAB, single antigen bead.

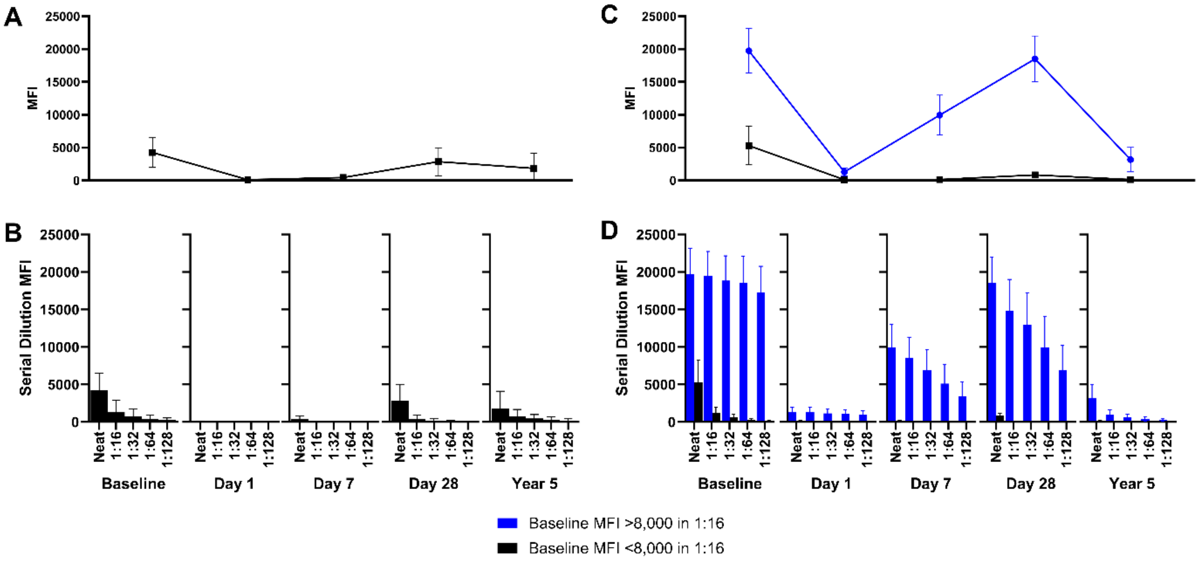

Figure S14: Participant 7 third-party antibodies with titrations. A) and C) show the HLA Class I and Class II third-party antibodies stratified by antibodies with MFI >8,000 in 1:16 dilution (likely complement-fixing) at baseline. B) and D) show HLA Class I and Class II third-party antibody serial dilution titrations, stratified by the same criteria. HLA, human leukocyte antigen; MFI, mean fluorescence intensity.

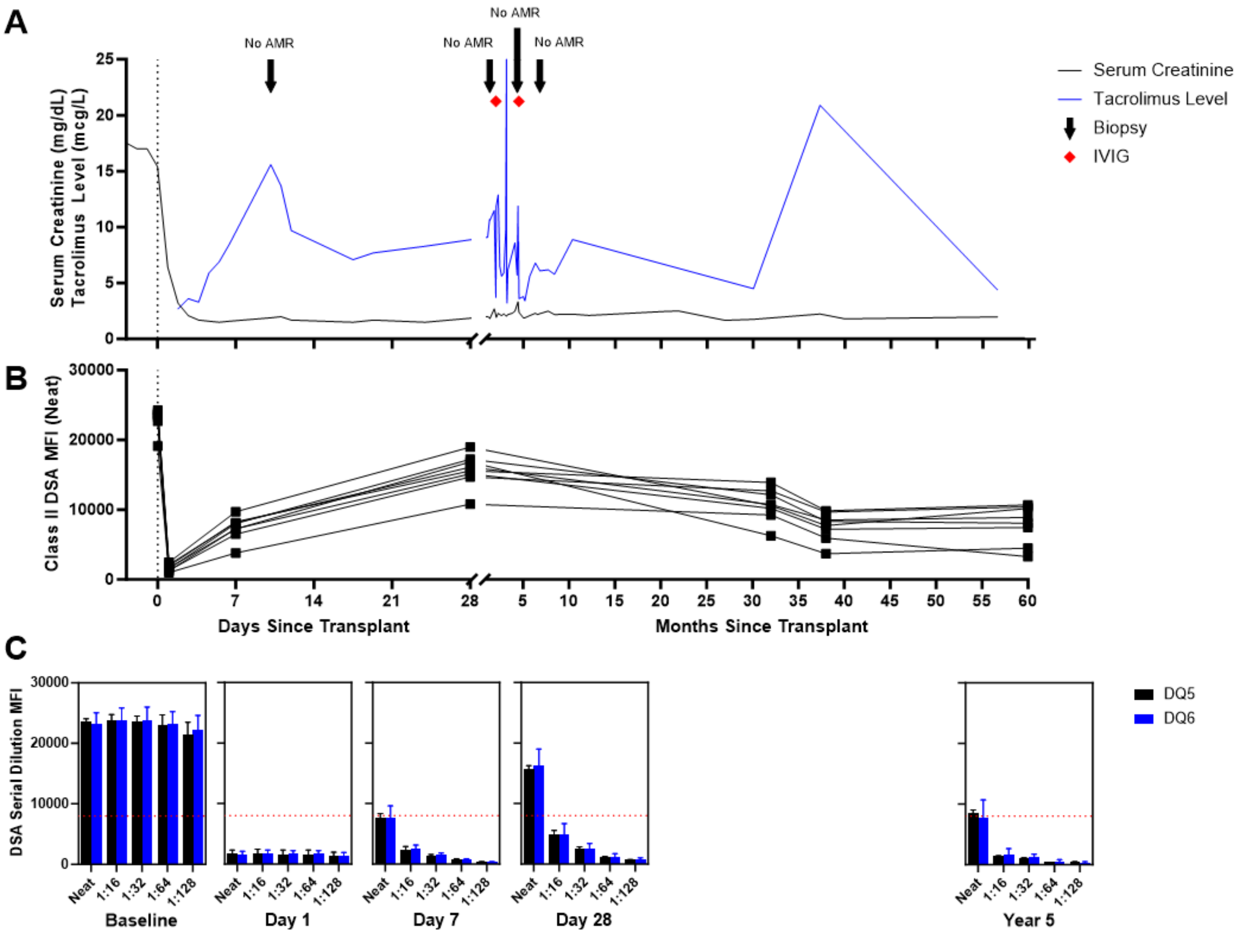

Figure S15. Clinical outcomes and DSA levels with titrations for Participant 8. A) shows the participant's serum creatinine over time, biopsy results, and immunomodulating treatments. B) shows DSA MFIs for each SAB. Panels A and B are presented on a common axis with the first 28 days presented in detail (left) followed by the ensuing 59 months of post-transplant follow-up (right). C) shows median MFI and standard deviation of DSA titrations performed by serial dilution, with a red dotted line at 8,000 MFI. AMR, antibody mediated rejection; DSA, donor specific antibodies; IVIG, intravenous immunoglobulin; MFI, mean fluorescence intensity; SAB, single antigen bead.

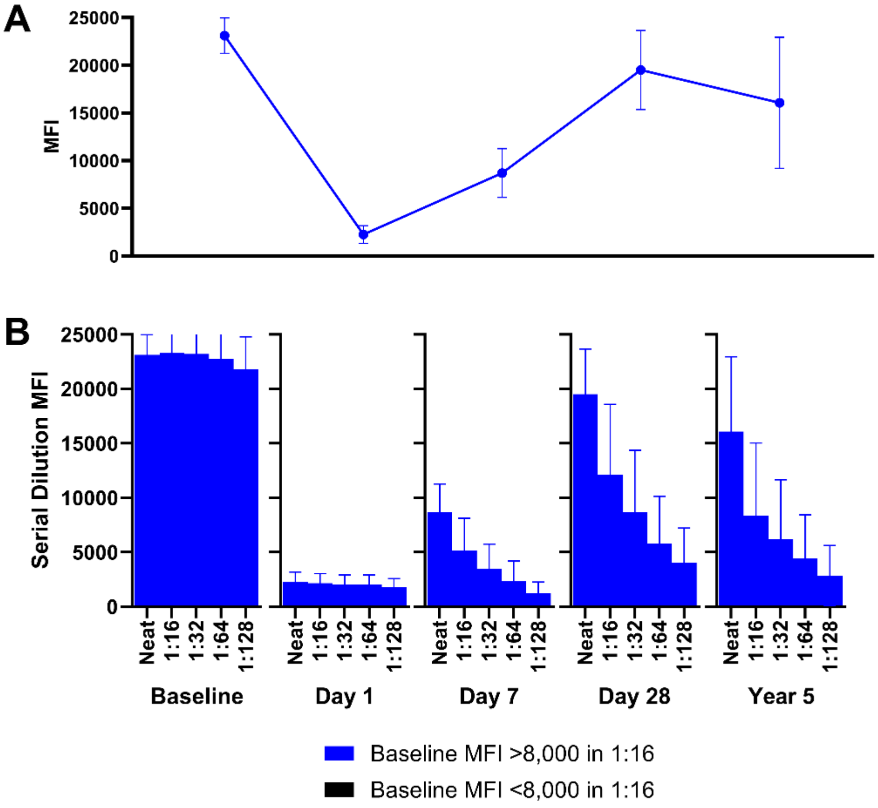

Figure S16. Participant 8 third-party antibodies with titrations. A) shows the HLA Class II third-party antibodies stratified by antibodies with MFI >8,000 in 1:16 dilution (likely complement-fixing) at baseline. B) shows HLA Class II third-party antibody serial dilution titrations, stratified by the same criteria. This participant did not have any third-party HLA Class I antibodies (MFI > 2,000 at any dilution). HLA, human leukocyte antigen; MFI, mean fluorescence intensity.

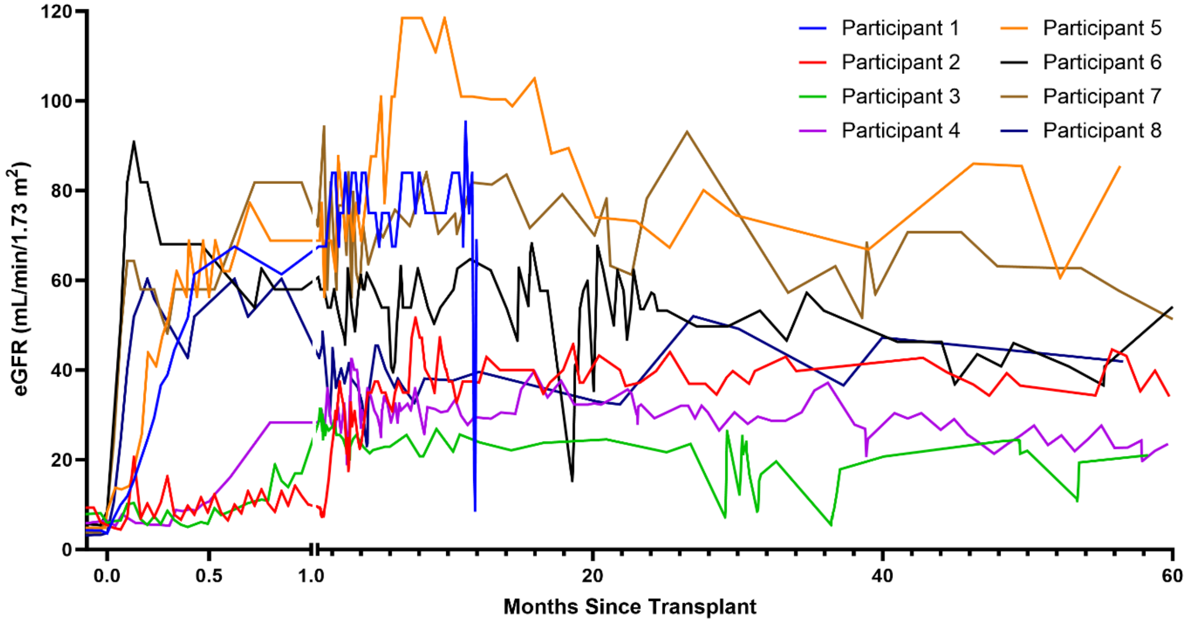

Figure S17. eGFR pre- and post-transplant. Estimated glomerular filtration rate (eGFR) was monitored as part of the original study protocol and then as part of routine clinical care.

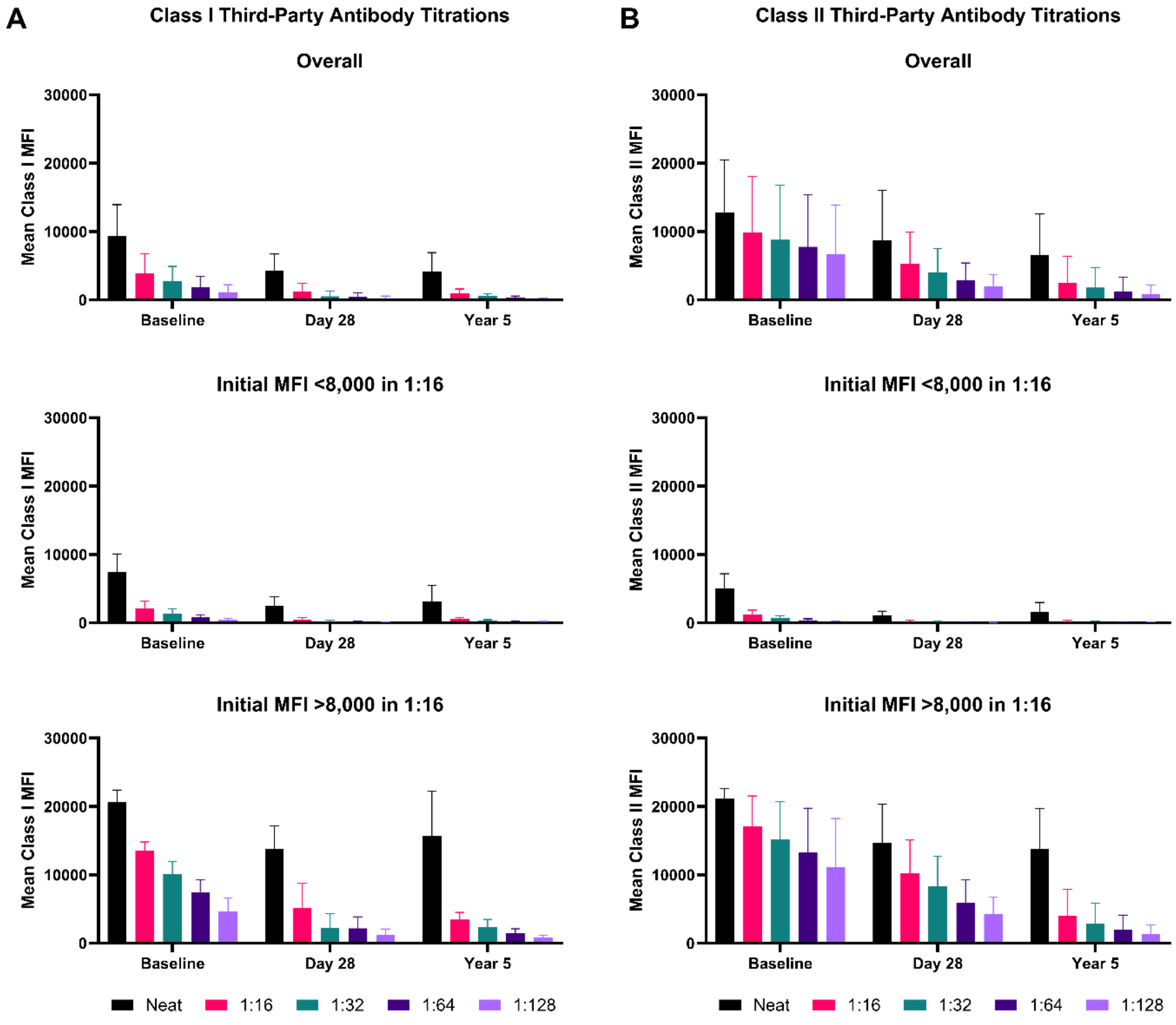

Figure S18. Aggregates of dilutional titrations for third-party antibody strength. Sera were serially diluted up to 1:128 concentration and separately run on Luminex® single antigen beads. Third-party antibodies were identified based on patient and donor HLA typing. A) shows HLA Class I third-party antibody titrations while B) shows HLA Class II third-party antibody titrations. In both cases, the upper panel shows the average MFI when all DSAs were included, while the middle panel shows the average MFI in each serial dilution restricted only to baseline weak third-party antibodies (pre-transplant MFI <8,000 in 1:16 dilution) and the lower panel shows the average MFI in each serial dilution restricted only to baseline strong third-party antibodies (pre-transplant MFI >8,000 in 1:16 dilution).

Supplementary Tables

Table S1: Renal Biopsies and Banff Scoring		
Participant	Biopsy Timing	Reported Banff Scoring
1	Day 12	g0-1, cg0, mm1, t0, ct0, i0, v0, cv0, ah0, aah0, ptc0-1, c4d3
	Month 6	g2, t0, i0, v0, ptc1-2
2	Day 6	g1, cg0, mm0, t0, ct0, i0, ci1, v0, cv0, ah0, ptc1, c4d2
	Day 10	g2, cg0, mm0, t0, ct0-1, i0, ci1, v0, cv2, ah1, ptc2, c4d3
	Day 17	g2, cg0, mm0, t0, i3, v0, cv0, ah1, ptc3, c4d3
	Day 25	g1, cg0, mm0, t0, i3, v0, cv0, ah1, ptc2-3, c4d3
	Month 2	g1, cg0, mm0, t0, ct1, i0-1, ci1, v0, cv0, ah0, ptc2, c4d3
	Month 4	g1, cg0, mm0, t0, ct1, i0, ci1, ah2, ptc1, c4d3
3	Day 15	g1, cg0, mm0, t0, ct0, i0, ci1, v0, cv0, ah2, ptc1, c4d0
	Day 20	g1, cg0, mm0, t1, ct0-1, i1, ci0, v0, cv1, ah1, ptc1, c4d0
	Day 26	g1, cg0, mm0, t2, i2-3, v0-1, cv2, ah1, ptc2, c4d0
	Month 2	g1, cg0, mm1, t0, ct1, i1, ci1-2, v0, cv2-3, ah2, ptc1, c4d0
	Month 3	g0, cg0, mm0, t0, ct0, i0-1, ci1-2, v0, cv1, ah0, ptc0, c4d0
	Month 6	g0-1, cg0, mm0, t0, ct1-2, i0, ci1-2, v0, cv1, ah0, ptc0, c4d0
	Month 9	g1, cg0, mm0, t0, ct1, i0, ci2, v0, cv1, ah2, ptc2, c4d0
4	Day 8	g1, cg0, mm0, t0, ct0, i0, ci0, v0, cv0, ah0, ptc2, c4d0
	Month 4	g0, cg0, mm0, t0-1, ct2, i1-2, ci2, v0, cv2, ah0, ptc2, c4d0
	Month 4	g0, cg0, mm0, t0-1, ct1, i1, ci1-2, v0, cv2, ah0, ptc2, c4d0
	Month 7	g0, cg0, mm0, t1, ct0, i1, ci1, v0, cv2, ah0, ptc1, c4d0
	Month 25	g2, cg1, mm1, t0, ct1, i1, ci2, ti2, v0, cv1, ah1, aah0, ptc1, c4d1
	Month 59	g0-1, t0, i0, v0, ptc1, c4d0. cg1, mm3, ct2, ci3, ti3, i-ifta1-2, cv2, ah2, aah0
5	Month 7	g1, cg 0, mm0, t1, ct1, i1, ci1, v0, cv0, ah0, ptc1-2, c4d0
6	Month 7	g0-1, cg0, mm2, t0, ct0, i0, ci0, v0, cv0, ah1, ptc1-2, c4d0
7	Day 8	g0, cg0, mm0, t0, ct0, i0, ci1, v0, cv0, ah0, ptc0, c4d0
	Month 6	g0, cg0, mm1, t0, ct0, i0, ci0, v0, cv0, ah0, ptc0, c4d3
	Month 15	g0, cg0, mm1, t1, ct1, i2, ci2-3, v0, cv2, ah0, ptc1, c4d2
8	Day 11	g0, cg0, mm0, t1, ct2, i0-1, ci1-2, v0, ah0, ptc1, c4d0
	Month 2	g0-1, cg0, mm0, t0, ct0, i0, ci1, v0, cv1, ah2, ptc1-2, c4d0
	Month 5	g0, cg0, mm0, t1, i3, v0, cv1, ah2, ptc1-2, c4d0
	Month 7	g0, cg0, mm1, t0, ct0, i0, ci1, v0, ah0, ptc1, c4d0
